# RAB7 deficiency impairs pulmonary artery endothelial function and promotes pulmonary hypertension

**DOI:** 10.1172/JCI169441

**Published:** 2024-02-01

**Authors:** Bryce Piper, Srimathi Bogamuwa, Tanvir Hossain, Daniela Farkas, Lorena Rosas, Adam C. Green, Geoffrey Newcomb, Nuo Sun, Jose A. Ovando-Ricardez, Jeffrey C. Horowitz, Aneel R. Bhagwani, Hu Yang, Tatiana V. Kudryashova, Mauricio Rojas, Ana L. Mora, Pearlly Yan, Rama K. Mallampalli, Elena A. Goncharova, David M. Eckmann, Laszlo Farkas

**Affiliations:** 1 Division of Pulmonary, Critical Care and Sleep Medicine, Department of Internal Medicine,; 2 Davis Heart and Lung Research Institute,; 3 Department of Anesthesiology, and; 4 Department of Cell Biology and Physiology, The Ohio State University (OSU), Columbus, Ohio, USA.; 5 Department of Physiology, Ziauddin University, Karachi, Pakistan.; 6 Linda and Bipin Doshi Department of Chemical and Biochemical Engineering, Missouri University of Science and Technology, Rolla, Missouri, USA.; 7 University of Pittsburgh, Heart, Blood, and Vascular Medicine Institute, Pittsburgh, Pennsylvania, USA.; 8 Division of Hematology, Department of Internal Medicine and The James Cancer Center, OSU, Columbus, Ohio, USA.; 9 Division of Pulmonary, Critical Care and Sleep Medicine, Department of Internal Medicine, University of California Davis, Davis, California, USA.; 10 Center for Medical and Engineering Innovation, OSU, Columbus, Ohio, USA.

**Keywords:** Pulmonology, Autophagy, Cellular senescence, Endothelial cells

## Abstract

Pulmonary arterial hypertension (PAH) is a devastating and progressive disease with limited treatment options. Endothelial dysfunction plays a central role in the development and progression of PAH, yet the underlying mechanisms are incompletely understood. The endosome-lysosome system is important to maintain cellular health, and the small GTPase RAB7 regulates many functions of this system. Here, we explored the role of RAB7 in endothelial cell (EC) function and lung vascular homeostasis. We found reduced expression of RAB7 in ECs from patients with PAH. Endothelial haploinsufficiency of RAB7 caused spontaneous pulmonary hypertension (PH) in mice. Silencing of RAB7 in ECs induced broad changes in gene expression revealed via RNA-Seq, and RAB7-silenced ECs showed impaired angiogenesis and expansion of a senescent cell fraction, combined with impaired endolysosomal trafficking and degradation, suggesting inhibition of autophagy at the predegradation level. Furthermore, mitochondrial membrane potential and oxidative phosphorylation were decreased, and glycolysis was enhanced. Treatment with the RAB7 activator ML-098 reduced established PH in rats with chronic hypoxia/SU5416. In conclusion, we demonstrate for the first time to our knowledge the fundamental impairment of EC function by loss of RAB7, causing PH, and show RAB7 activation to be a potential therapeutic strategy in a preclinical model of PH.

## Abstract 2

<p>Reduced endothelial expression of endolysosomal RAB7 causes dysregulation of autophagy, mitochondrial dysfunction, endothelial senescence, and induces spontaneous pulmonary hypertension.</p>

## Graphical abstract

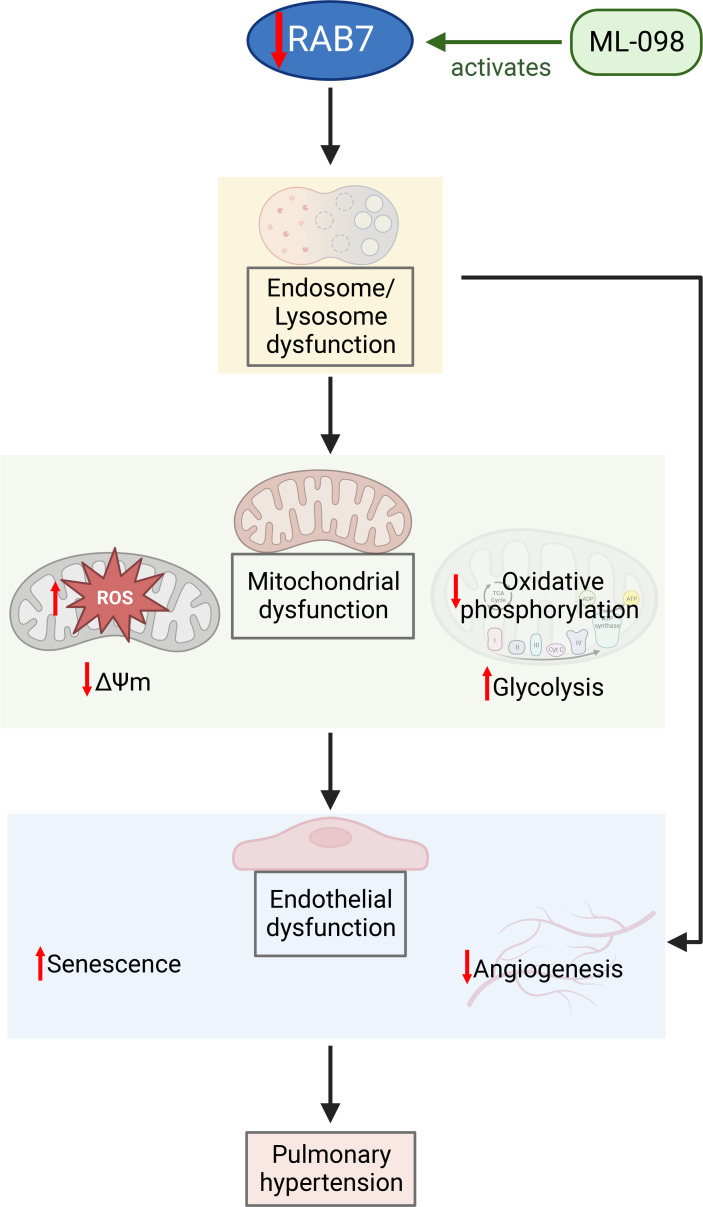

## Introduction

Pulmonary arterial hypertension (PAH) is a severe and fatal condition characterized by increased pulmonary artery (PA) pressure and extensive remodeling of all layers of the PA wall (1, 2). The molecular pathogenesis of PAH remains incompletely understood at this time (1). One important and defining feature of pulmonary arterial dysfunction and remodeling in PAH is lung endothelial cell (EC) dysfunction. Following endothelial injury and apoptosis, endothelial dysfunction presents as unchecked proliferation that likely follows endothelial injury and apoptosis, impaired angiogenesis, enhanced release of proinflammatory cytokines, and upregulation of adhesion molecules (1). Similar to cancer, the hyperproliferative ECs in PAH may derive from clonal expansion of primitive stem cell–like ECs (3, 4). Our previous work indicates also that, while these primitive, hyperproliferative ECs can promote lung vascular remodeling, this remodeling is reversible after the removal of additional triggers, such as hypoxia (4). This result indicates that proliferation alone may not be sufficient to develop progressive pulmonary vascular remodeling and PAH. Recently, a subpopulation of senescent ECs was identified in PA endothelial cells (PAECs) from patients with several forms of pulmonary hypertension (PH), including PAH, and these senescent ECs appear to promote the irreversibility of PA remodeling and PH (5, 6). We will use the term “PH” when describing animal models throughout the manuscript. The dysfunctional ECs from patients with PAH further exhibit dysfunction of mitochondria, including dysregulation of mitochondrial membrane potential ΔΨ_m_, reduced mitochondrial mass, impaired oxidative phosphorylation, and glycolytic shift akin to that seen in cancer cells (7–10). PAH ECs show reduced oxidative phosphorylation, but the impaired regulation of mitochondrial health in PAH ECs is not sufficiently explained (8). Key cellular functions are regulated by autophagy, or the endolysosomal degradation of macromolecules and cell organelles, including mitochondria (11). In animal models of PH, conflicting results either attribute a protective function to physiologic autophagy or indicate that inhibition of aberrant autophagy is a treatment for PH (12, 13). Endosomal sorting, trafficking, and fusion of endosomes and lysosomes to endolysosomes are critical steps in physiologic autophagy (14). The small GTPase RAB7A has a key function in all these steps (15, 16). RAB7A is commonly referred to as RAB7, which we will use throughout the manuscript. Consequently, loss of RAB7 disrupts cellular function and impairs mitochondrial health, amplifying tissue injury (17–20).

We hypothesized that endothelial RAB7 deficiency is a cause of endothelial dysfunction in PAH through inhibition of autophagy on the predegradation level and promotes lung vascular remodeling and PH. We show here, for the first time to our knowledge, reduced RAB7 expression in PAECs from patients with PAH and found that endothelium-specific reduction of RAB7 expression caused spontaneous PH in mice. In vitro, RAB7 knockdown impaired endolysosomal trafficking and degradation and fundamentally altered gene expression in PAECs with functional evidence of impaired angiogenesis and expansion of a senescent EC subpopulation. In RAB7-deficient ECs, we detected a loss of ΔΨ_m_, with a shift toward dysfunctional mitochondria and evidence of reduced mitochondrial respiration with enhanced glycolysis. The RAB7 GTPase activator ML-098 reduced severe PH in chronic hypoxia/SU5416 (Hx/Su) rats, thus identifying RAB7 as a potential therapeutic target in PAH.

## Results

### Endosomal GTPase RAB7 is reduced in PAECs from patients with PAH.

**Figure 1:**
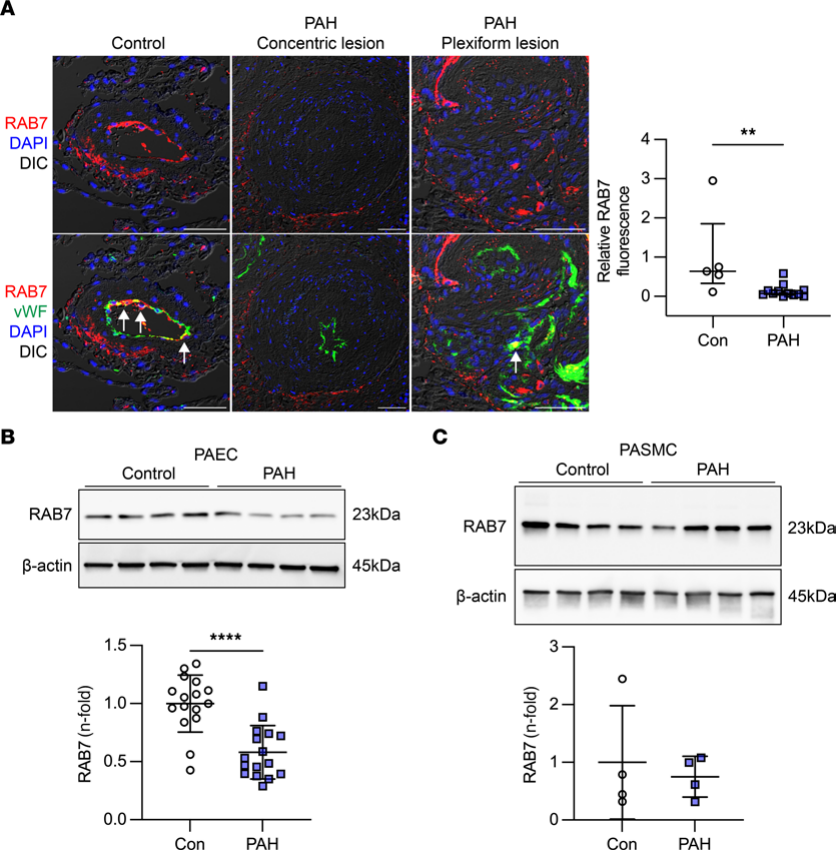
Reduced RAB7 expression in endothelial cells from PAH patients. (**A**) Representative optical sections (confocal microscopy, representative of 5 PAs from 3 control [Con] patients and 12 PAs from 3 patients with PAH) show reduced RAB7 expression in ECs in concentric and plexiform lesions from patients with PAH compared with controls. Arrows indicate vWF^+^ ECs with strong RAB7 expression. Scale bars: 50μm. Nuclei were stained with DAPI. The graph shows quantification of relative RAB7 immunofluorescence in ECs from control and PAH PAs. (**B** and **C**) Representative Immunoblot (*n* = 4 individual control and PAH PAEC lines) and quantification of RAB7 in PAECs (**B**) in 4 immunoblots from 12 individual controls (failed donors) and 15 individual patients with PAH (*n* = 16 data points total per group from 4 repeat experiments). (**C**) Representative immunoblot and quantification of RAB7 in PASMCs from 4 individual controls (failed donors) and 4 individual patients with PAH. β-Actin was used as the loading control. All graphs show single values and the median ± IQR (**A**) or the mean ± SD (**B** and **C**). **P < 0.01 and ****P < 0.0001, by 2-tailed Mann-Whitney *U* test (**A**) or 2-tailed Student’s *t* test (**B** and **C**).

We first identified the expression of RAB7 in lung tissues, PAECs, and PA smooth muscle cells (PASMCs) from patients with PAH. The expression of RAB7 was reduced in vWF^+^ PAECs in concentric and plexiform lesions from patients with PAH compared with control lungs (Figure 1A). PAH PAECs had reduced expression of RAB7, whereas PAH PASMCs had RAB7 protein expression levels similar to those of control PAECs (Figure 1, B and C). Supplemental Tables 1–3 provide the demographic information for the tissues and cell lines; supplemental material available online with this article; https://doi.org/10.1172/JCI169441DS1

### Endothelial RAB7 haploinsufficiency causes spontaneous PH.

**Figure 2:**
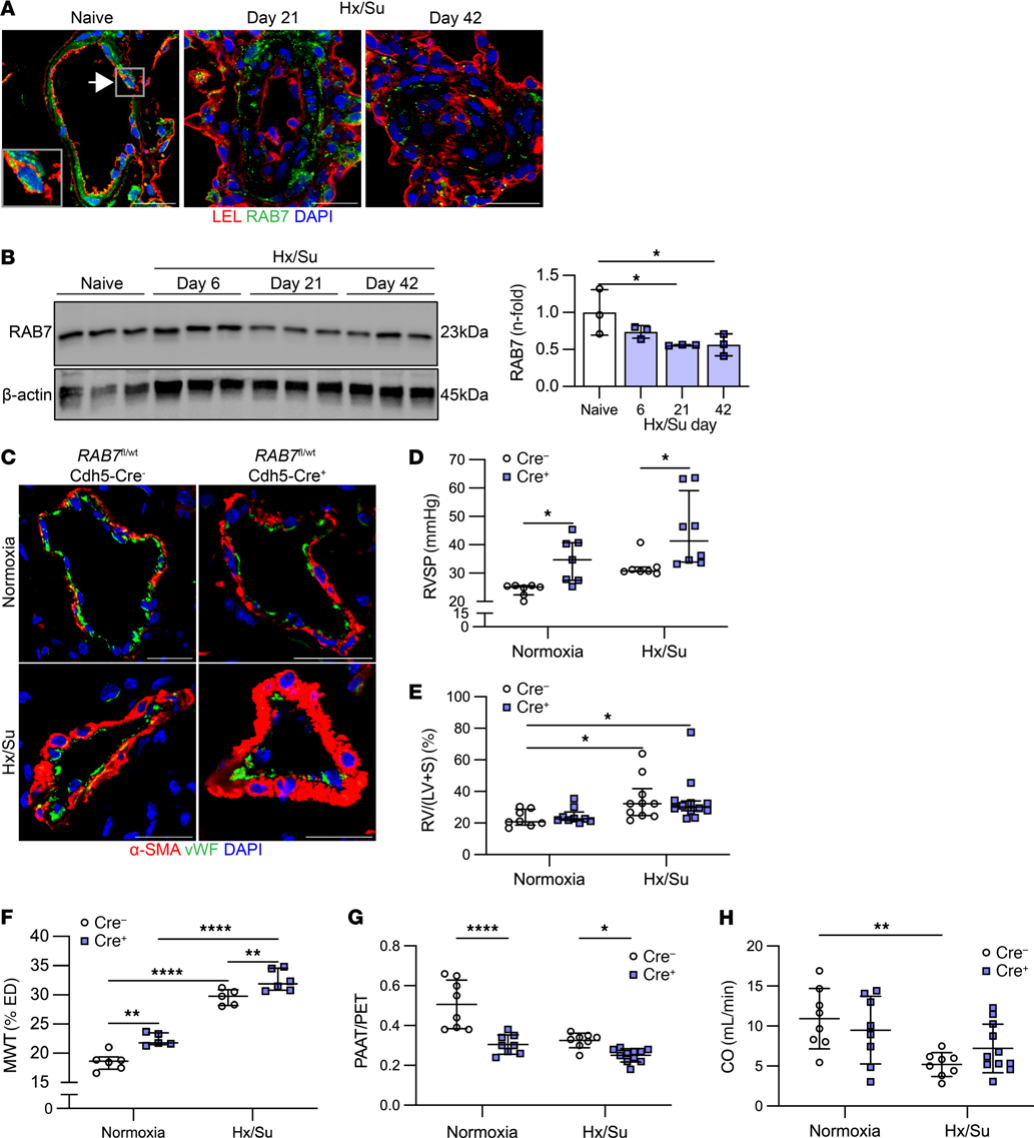
Loss of RAB7 expression causes PH in vivo. (**A**) Representative confocal microscopic immunofluorescence images (representative of 3 animals per group) show strong RAB7 staining (green pseudocolor) in PAs from naive rats, including in PAECs (a representative cell is indicated by the arrow and is shown in more detail in the inset). PAECs are indicated by *Lycopersicon* *esculentum*, tomato lectin (LEL) staining (red pseudocolor). In Hx/Su-treated rats, RAB7 expression decreased in ECs in the remodeled PAs at days 21 and 42. Scale bars: 25 μm. Original magnification, ×600 (insets). (**B**) Representative immunoblot and densitometric analysis of RAB7 expression in naive and Hx/Su rats. (**C**) Representative double immunofluorescence for vWF and α-SMA (optical section, confocal microscopy). Scale bars: 25 μm. (**D**–**H**) RVSP (**D**), Fulton RV hypertrophy index: RV/(left ventricle + septum) [RV/(LV+S)] (**E**), PA MWT (**F**), PAAT/PET (**G**), echocardiographically estimated cardiac output (CO) (**H**) of *RAB7^fl/WT^* Cdh5-Cre^-^ and *RAB7^fl/WT^* Cdh5-Cre^+^ mice exposed to normoxia and Hx/Su. *n* = 3 per group (**B**); *n* = 7, except *n* = 8 for Hx/Su Cre^+^ (**D**); *n* = 8, except *n* = 9 (normoxia Cre^+^), *n* = 10 (Hx/Su Cre^-^ and *n* = 12 (Hx/Su Cre^+^) (**E**); *n* = 5 (normoxia Cre^+^ and Hx/Su Cre^–^), *n* = 6 (normoxia Cre^-^ and Hx/Su Cre^+^) (**F**); *n* = 8, except *n* = 12 (Hx/Su Cre^+^) (**G** and **H**). *P < 0.05, **P < 0.01, and ****P < 0.0001, by 1-way ANOVA followed by Holm-Šidák multiple-comparison test (**B**), 2-way ANOVA with Holm-Šidák multiple-comparison test, and evaluation of normality of residual distribution (D’Agostino-Pearson) (**D** and **F**), and Kruskal-Wallis analysis with Dunn’s multiple-comparison test (**E**). All graphs show single values and the mean ± SD (**B**, **F**, **G**, and **H**) or the median ± IQR (**D** and **E**).

We then studied RAB7 expression in the chronic Hx/Su rat model of PH, because this model reproduces many features of PAH, including the occlusive pulmonary arteriopathy that is characteristic of PAH (21, 22). Using lung tissue sections and protein lysates, we found an overall reduction of RAB7 expression in the lung tissue of Hx/Su rats at days 21 and 42 and that this reduction was localized to ECs in the remodeled PAs of Hx/Su rats (Figure 2, A and B). Then, we generated heterozygous EC–specific *RAB7*-KO (endothelial *RAB7* haploinsufficient) mice by crossbreeding *RAB7^fl/fl^* mice with Cdh5-Cre mice. We found increased medial wall thickness (MWT) and right ventricular systolic pressure (RVSP) and a decreased ratio of PA acceleration time/pulmonary ejection time (PAAT/PET) in *RAB7^fl/WT^* Cdh5-Cre^+^ mice after exposure to Hx/Su, indicating exaggerated PA remodeling and PH with endothelial *RAB7* haploinsufficiency (Figure 2, C–G). Echocardiographically estimated right ventricular (RV) cardiac output was reduced in Hx/Su-exposed *RAB7^fl/WT^* Cdh5-Cre^–^ mice, with no difference observed between Cre^–^ and Cre^+^ mice (Figure 2H). We further evaluated the RV changes and found that endothelial *RAB7* haploinsufficiency impaired RV capillary density (Supplemental Figure 1) without affecting the RV hypertrophic response, as measured by the Fulton index and the RV cardiomyocyte (CM) cross-sectional area (CSA) (Figure 2E and Supplemental Figure 2). Further, we examined whether endothelial *RAB7* haploinsufficiency promotes endothelial-mesenchymal transition (EnMT) in vivo. We analyzed the presence of ECs expressing the mesenchymal/smooth muscle cell marker α–smooth muscle actin (α-SMA) and of ECs with nuclear expression of the EnMT transcription factor SNAIL. While Hx/Su treatment increased the fraction of α-SMA^+^ ECs and SNAIL^+^ ECs, endothelial *RAB7* haploinsufficiency promoted endothelial SNAIL expression in PAs following Hx/Su exposure, but not under normoxic conditions (Supplemental Figure 3).

### Gene silencing of RAB7 induces endothelial dysfunction in PAECs.

**Figure 3:**
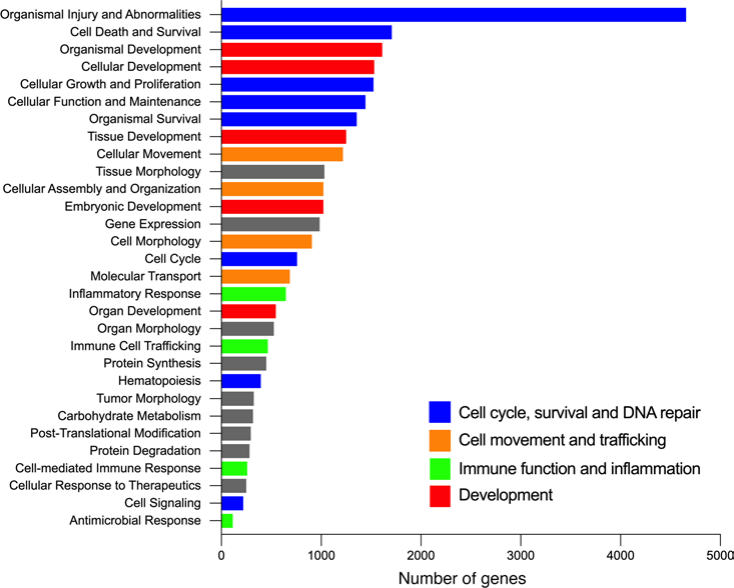
RNA-Seq of *RAB7*-silenced PAECs. Ingenuity Pathway Analysis of bulk RNA-Seq data in PAECs transfected with *RAB7* siRNA versus control siRNA (fold change >|1.25|, adjusted *P* value <0.05) (representing *n* = 3 per group). The diagram lists the 30 most regulated function terms and shows the number of DEGs in the RNA-Seq data set within each category. DEG terms were labeled according to their relevance for cell-cycle and DNA repair (blue), cellular movement and trafficking (orange), immune function and inflammation (green), and development (red).

**Figure 4:**
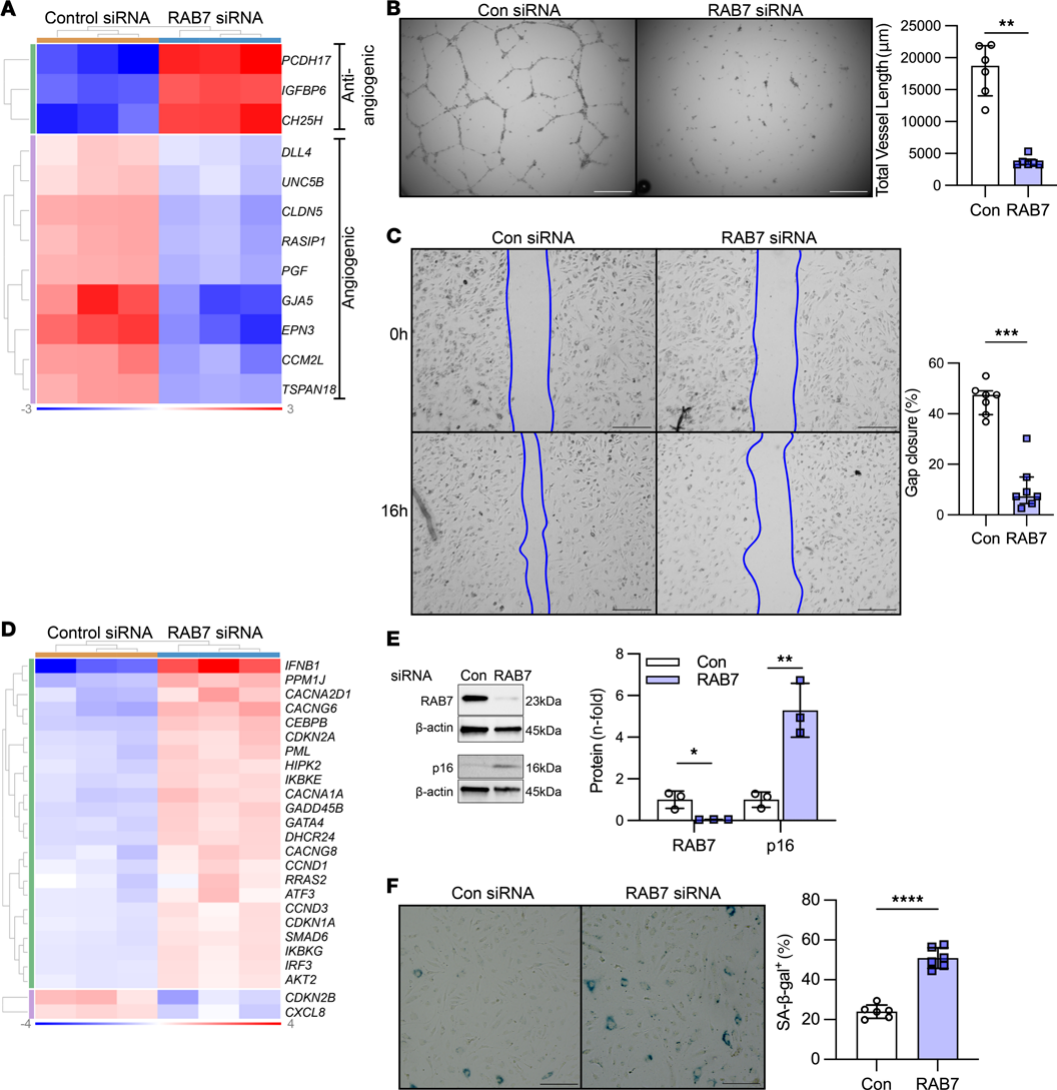
Loss of RAB7 induces endothelial dysfunction and senescence in PAECs. (**A**) The clustered heatmap shows increased expression of antiangiogenic genes and reduced expression of angiogenic and EC barrier function genes in RNA-Seq of PAECs after *RAB7* siRNA treatment versus treatment with control siRNA. *n* = 3 per group. (**B**) Representative phase-contrast images after 24 hours and quantification of total network length in *RAB7* siRNA–treated PAECs. *n* = 6 per group. (**C**) Representative phase-contrast images after 16 hours of a gap closure assay and quantification of the percentage of gap closure in *RAB7* siRNA–transfected PAECs versus treatment with control siRNA. *n* = 7 per group. (**D**) Clustered heatmap of DEGs of senescence-associated gene expression pattern (Ingenuity Pathway Analysis) in RNA-Seq of PAECs treated with *RAB7* siRNA. *n* = 3 per group. (**E**) Representative immunoblots and densitometric quantification for RAB7 and p16 in PAECs treated with *RAB*7 siRNA. *n* = 3 per group. (**F**) Representative images and quantification of the fraction of SA–β-gal^+^ PAECs after *RAB7* siRNA treatment versus treatment with control siRNA. *n* = 6 per group. Data are shown as single values and the median ± IQR (**B** and **C**) or the mean ± SD (**E** and **F**). *P < 0.05, **P < 0.01, ***P < 0.001, ****P < 0.0001, by 2-tailed Mann-Whitney *U* test (**B** and **C**) or 2-tailed Student’s *t* test (**E** and **F**). Heatmap data are normalized log_2_ fold expression. Data in **B**, **C**, **E**, and **F** are representative of 2 or more experiments. Scale bars: 250 μm (**B** and **C**) and 200 μm (**F**).

We then tested whether RAB7 expression contributes to physiological EC function. We performed siRNA targeting of *RAB7* and bulk RNA-Seq and found that *RAB7* knockdown resulted in differential expression of 4,842 genes using analysis parameters of a fold change >|1.25| and an adjusted *P* < 0.05. We detected a substantial amount of differentially expressed genes (DEGs) with function in cell-cycle and DNA repair, cellular movement and trafficking, immune function and inflammation, and development (Figure 3). Genes affecting angiogenesis and EC barrier function were among the DEGs with the highest *n*-fold changes in *RAB7* siRNA–treated PAECs. We detected upregulation of the antiangiogenic genes *PCDH17*, *IGFBP6*, and *CH25H* and downregulation of the angiogenic and EC function genes *DLL4*, *UNC5B*, *CLDN5*, *RASIP1*, *PGF*, *GJA5*, *EPN3*, *CCM2L*, and *TSPAN18* (Figure 4A). We then tested EC function in vitro and found that knockdown of *RAB7* inhibited angiogenic network formation and gap closure, indicating impaired migration (Figure 4, B and C). Consistent with these functional changes, our RNA-Seq data revealed a prosenescent transcriptomic signature in *RAB7* siRNA–treated PAECs (Figure 4D). These findings were mirrored by elevated p16 protein levels (Figure 4E) and an increased fraction of senescence-associated β-galactosidase–positive (SA–β-gal^+^) PAECs (Figure 4F). We then tested the potential role of RAB7 deficiency in EnMT in vitro. *RAB7* knockdown induced the activation of Ingenuity pathways that promote fibrotic remodeling and EnMT (Supplemental Figure 4A). In addition, some changes in mRNA indicated an EnMT phenotype, as shown by the reduction of mRNA of cadherin 5 (*CDH5)* and elevation of transgelin (*TAGLN)* (Supplemental Figure 4B). At the protein level, the key finding was the downregulation of platelet endothelial cell adhesion molecule 1 (PECAM1), a marker of ECs. We did, however, not observe a concurrent upregulation of proteins typically associated with vascular smooth muscle cells, such as α-SMA, calponin 1, and SM-22α (TAGLN). Furthermore, our analysis did not reveal a statistically significant upregulation of snail family transcriptional repressor 1 (SNAIL), a transcription factor often implicated in the regulation of EnMT (Supplemental Figure 4, C–E).

### RAB7 is required for endolysosome function in PAECs.

**Figure 5:**
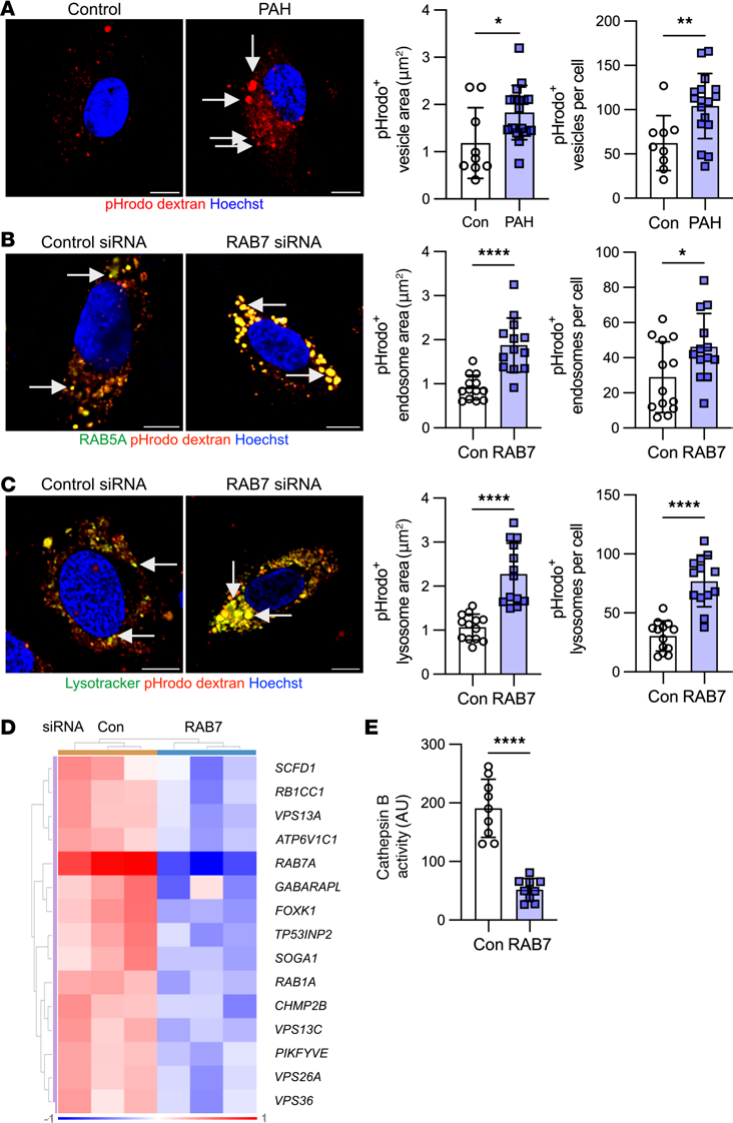
Impaired endosome-lysosome function in PAH PAECs and RAB7-deficient PAECs. (**A**) Representative optical sections (confocal microscopy) and quantification of pHrodo dextran^+^ vesicle area and number of vesicles per cell indicate the accumulation of pHrodo dextran after 20 minutes in enlarged vesicles in PAH PAECs (arrows), but not in control PAECs. pHrodo dextran is taken up by endosomes and emits red fluorescence signal when the pH drops during endosomal acidification. *n* = 9 (control) and *n* = 17 (PAH). (**B**) Representative optical sections (confocal microscopy) show an accumulation of pHrodo dextran after 20 minutes in enlarged early endosomes with *RAB7* silencing. Early endosomes were identified by transfection of PAECs with baculovirus expressing GFP-labeled RAB5. Quantification of RAB5^+^ pHrodo dextran^+^ endosome area and number per cell confirmed that dextran accumulated in enlarged early endosomes following RAB7 silencing. *n* = 13 per group. (**C**) Representative optical sections (confocal microscopy) show an accumulation of pHrodo dextran after 20 minutes in enlarged lysosomes in *RAB7* siRNA–treated PAECs. Lysosomes were labeled with LysoTracker. Quantification of LysoTracker^+^ pHrodo dextran^+^ lysosome area and number per cell confirmed that dextran accumulated in enlarged lysosomes following RAB7 silencing. *n* = 13 per group. (**D**) Clustered heatmap shows autophagy-related DEGs that were found to be downregulated in RNA-Seq from PAECs treated with *RAB7* siRNA. Expression is normalized log_2_-fold. (**E**) Reduced cathepsin B activity also indicates impaired lysosomal autophagy. *n* = 9 per group. Scale bars: 10 μm (**A**–**C**). All graphs show single values and the mean ± SD. Data in **A**–**C** are representative of 2 or more experiments. *P < 0.05, **P < 0.01, ****P < 0.0001, by 2-tailed Student’s *t* test.

Endolysosome function requires RAB7 (15, 23–25). To identify the extent to which RAB7 is required for endolysosome function in PAECs, we studied intracellular endolysosome trafficking in PAECs from patients with PAH and in control PAECs following *RAB7* knockdown. Using pHrodo dextran, which emits fluorescence after acidification in endosomed and lysosomed, we detected accumulation of dextran in PAH PAECs in enlarged vesicles (Figure 5A). Using baculovirus-mediated expression of the GFP-labeled RAB5A, a marker of the early endosome, we found that pHrodo dextran accumulated in enlarged RAB5A^+^ early endosomes following endosomal uptake in *RAB7*-silenced PAECs (Figure 5B). *RAB7* siRNA also caused the accumulation of pHrodo dextran in enlarged lysosomes, which we detected by staining with LysoTracker (Figure 5C). Consistently, our RNA-Seq data revealed the downregulation of multiple autophagy-related genes with *RAB7* siRNA (Figure 5D). Reduced cathepsin B activity further indicated impaired lysosomal autophagy in *RAB7*-silenced PAECs (Figure 5E).

### RAB7 knockdown impairs mitochondrial membrane potential and oxidative phosphorylation.

**Figure 6:**
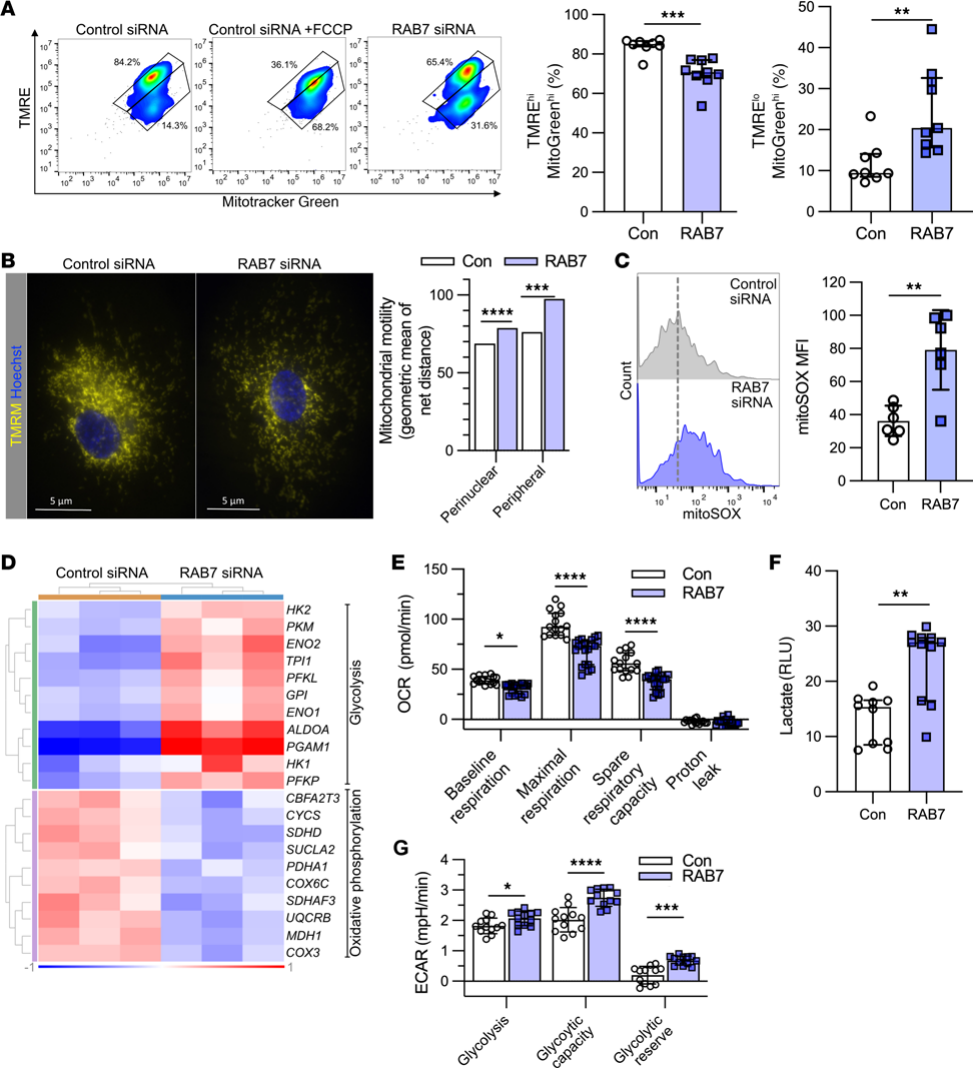
*RAB7* silencing impairs mitochondrial membrane potential and mitochondrial function. (**A**) *RAB7* siRNA impaired ΔΨ**_m_** as assessed by TMRE and MitoTracker Green (MitoGreen) flow cytometry. Carbonyl cyanide FCCP was used as a positive control (depolarizes mitochondrial membrane). TMRE^hi^ MitoGreen^hi^ cells indicate cells with functional mitochondria, whereas TMRE^lo^ MitoGreen^hi^ cells are cells with dysfunctional mitochondria. *n* = 8 (control) and *n* = 9 (RAB7). (**B**) Representative TMRM-stained images show overall reduction and perinuclear accumulation of functional mitochondria in *RAB7* siRNA–treated PAECs. Results are representative of 3 experiments. Scale bars: 5 μm. *RAB7* knockdown increased mitochondrial motility in peripheral and perinuclear regions. (**C**) *RAB7* knockdown promoted mitochondrial ROS production as indicated by flow cytometry for mitoSOX. *n* = 6 per group for the representative histogram plots and quantification of MFI. (**D**) Clustered heatmap shows upregulated glycolysis-related DEGs and downregulated oxidative phosphorylation–related DEGs in bulk RNA-Seq from PAECs plus *RAB7* siRNA. Expression is normalized log_2_-fold. (**E**) Seahorse high-resolution respirometrics show a reduced OCR with *RAB7* siRNA at basal respiration, maximal respiration, and spare respiratory capacity. *n* = 15 (control) and *n* = 21 (RAB7). (**F**) Luminescence assay for lactate: increased lactate production in *RAB7* siRNA–treated PAECs. *n* = 10 (control) and *n* = 11 (RAB7). (**G**) ECAR data reveal more rapid acidification (i.e., greater reliance on glycolysis) in the *RAB7* siRNA–treated PAECs as shown for glycolysis and the basal and maximum glycolytic rates. *n* = 12 per group. All graphs show single values and the median ± interquartile range (**A**, **E**, and **F**) or the mean ± SD (**C** and **G**) (except **B**, as bar graphs in **B** indicate the geometric mean of a log-normal distribution). Data in **A**–**C**, **E**, **F**, and **G** are from 2 or more experiments. *P < 0.05, **P < 0.01, ***P < 0.001, and ****P < 0.0001, by 2-tailed Mann-Whitney *U* test (**A** and **F**), 2-tailed Student’s *t* test (**C**), and 2-way ANOVA (**E** and **G**) with Holm-Šidák post hoc test and normality testing of residuals (D’Agostino-Pearson).

Because autophagy is important to maintain physiologic mitochondrial function (26), we tested the role of RAB7 in maintenance of the mitochondrial membrane potential ΔΨ_m_ and mitochondrial respiration. Using a flow cytometry assay for tetramethylrhodamine ethyl ester (TMRE) and MitoTracker Green, we found that *RAB7* knockdown reduced the fraction of ECs with functional mitochondria (polarized), while increasing the fraction of ECs with dysfunctional (depolarized) mitochondria, indicating an overall reduction in ΔΨ_m_ (Figure 6A). Using fluorescence microscopy of tetramethylrhodamine, methyl ester (TMRM) staining, *RAB7* silencing caused a net reduction in ΔΨ_m_ with accumulation of the remaining functional mitochondria in the perinuclear region (Figure 6B). We further detected increased mitochondrial net motility in perinuclear and peripheral regions in *RAB7* siRNA–treated PAECs (Figure 6B). *RAB7* silencing also increased mitochondrial production of ROS, as measured by a MitoSOX flow cytometry assay (Figure 6C). RNA-Seq analysis of PAECs treated with *RAB7* siRNA revealed upregulation of mRNA for multiple genes related to glycolysis, whereas the mRNA expression of various genes related to oxidative phosphorylation was reduced (Figure 6D). *RAB7* knockdown also impaired oxidative phosphorylation because our data showed a reduced oxygen consumption rate (OCR) at baseline, maximal respiration, and spare respiratory capacity (Figure 6E). There was no difference in proton leak. In addition, *RAB7* siRNA–treated PAECs had higher lactate production than control siRNA–treated PAECs, indicating a shift toward glycolysis (Figure 6F). Last, the extracellular acidification rate (ECAR) measurements revealed increased glycolysis, glycolytic capacity, and glycolytic reserve (Figure 6G).

### The RAB7 activator ML-098 reduces experimental PH in rats.

**Figure 7:**
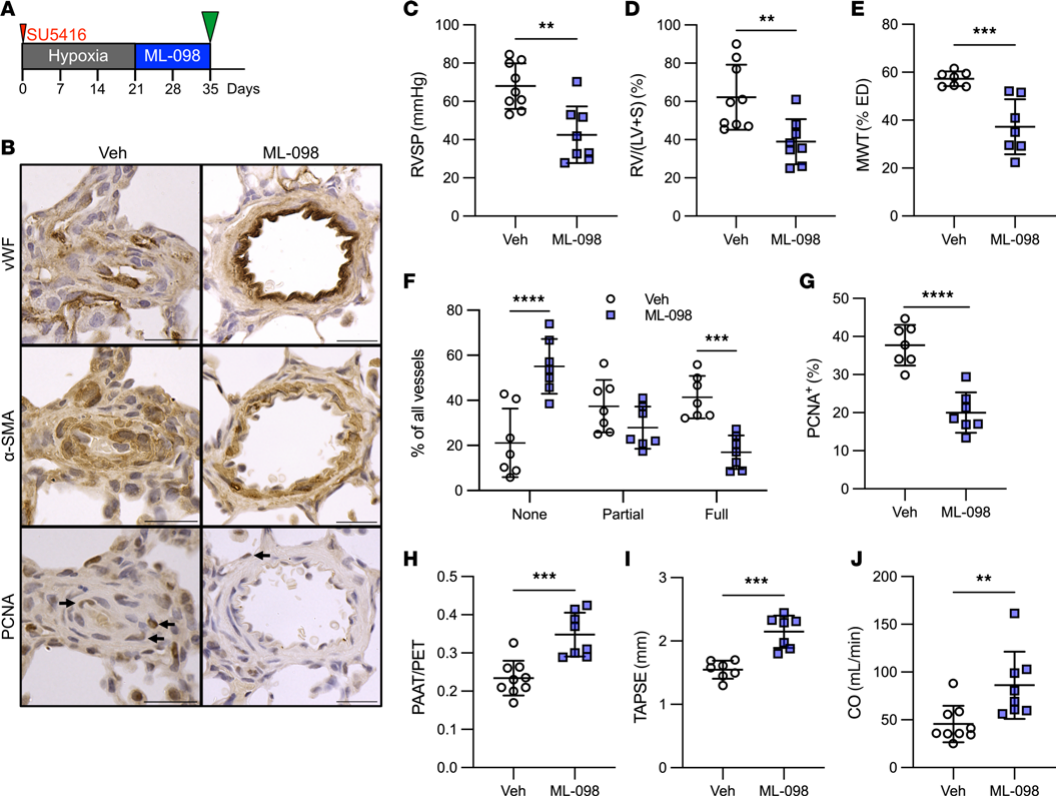
RAB7 activator ML-098 reduces PH in rats exposed to chronic hypoxia/SU5416. (**A**) Interventional treatment diagram. (**B**) Representative vWF, α-SMA, and PCNA immunohistochemistry showing serial sections of the same PA. (**C**) RVSP, (**D**) Fulton index, (**E**) MWT of small PAs, (**F**) occlusion of small PAs, (**G**) percentage of PCNA^+^ PA mural cells, (**H**) ratio of PAAT versus PET, (**I**) TAPSE, and (**J**) echocardiographic estimation of RV CO. Scale bars: 25 μm. *n* = 9 (vehicle) and *n* = 8 (ML-098) (**C**, **D**, **H**, and **J**), *n* = 7 (**E**–**G** and **I**). All graphs show single values and the mean ± SD. **P < 0.01, ***P < 0.001, and ****P < 0.0001, by 2-sided Student’s *t* test. Veh, vehicle.

**Figure 8:**
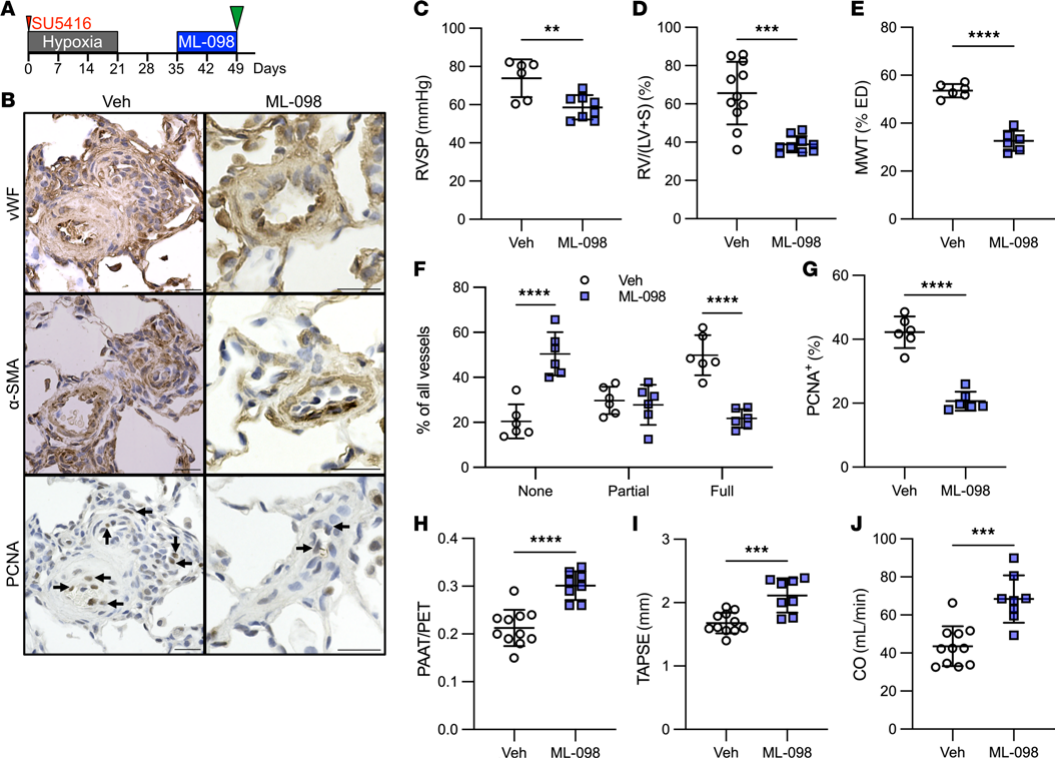
RAB7 activator ML-098 reverses established PH in rats exposed to Hx/Su. (**A**) Reversal treatment diagram. (**B**) Representative vWF, α-SMA, and PCNA immunohistochemistry showing serial sections of the same PA. (**C**) RVSP, (**D**) the Fulton index, (**E**) MWT of small PAs, (**F**) occlusion of small PAs, (**G**) percentage of PCNA^+^ PA mural cells, and echocardiographic data for (**H**) the ratio of PAAT to PET, (**I**) TAPSE, and (**J**) RV CO. Scale bars: 25 μm. *n* = 6 (vehicle) and *n* = 8 (ML-098) (**C**), *n* = 11 (vehicle) and *n* = 9 (ML-098) (**D**), *n* = 6 (**E**–**G**), *n* = 11 (vehicle) and *n* = 8 (ML-098) (**H**–**J**). All graphs show single values and the mean ± SD. Data were analyzed using 2-sided Student’s *t* test. **P < 0.01, ***P < 0.001, and ****P < 0.0001.

To aid the translation of our findings to a potential treatment option, we tested whether the RAB7 GTPase activator ML-098 reduces experimental PH induced by Hx/Su in rats. First, we performed a preventive dose-response experiment (days 1–21) to find the lowest efficacious dose. We found overall that 1.0 and 10 mg/kg doses reduced PA occlusion, MWT, and RVSP most effectively and successfully increased PAAT/PET and cardiac output, which were determined by echocardiography (Supplemental Figure 5). Given these results, we opted for 1.0 mg/kg in the interventional treatment approach (days 22–35). In this approach, ML-098 treatment reduced RVSP, the Fulton index, MWT, PA occlusion, and mural cell proliferation, while increasing PAAT/PET, tricuspid annular plane systolic excursion (TAPSE), and cardiac output (Figure 7). In addition, our data also showed a reduction in EnMT in the PAs of Hx/Su rats treated with ML-098 compared with those of vehicle-treated Hx/Su rats as indicated by a reduced fraction of α-SMA^+^ ECs and SNAIL^+^ ECs in the PAs (Supplemental Figure 6). The results from the interventional treatment were further supported by our data from a reversal treatment approach with ML-098 (days 36–49). This treatment regimen resulted in a reduction of RVSP, the Fulton index, MWT, PA occlusion, and mural cell proliferation, and in an increase in PAAT/PET, TAPSE, and cardiac output (Figure 8). Hence, the RAB7 GTPase activator ML-098 prevented and reduced occlusive PA remodeling, EnMT, PH, and RV dysfunction induced by Hx/Su.

## Discussion

PAH remains a deadly disease, and current vasodilator therapies are not sufficient to cure it, given the lack of effect on the progressive occlusive pulmonary arteriopathy that is a hallmark of PAH (1, 2). One potential underlying cause is that these treatments mainly improve the vasotonus function of ECs but not the overall EC dysfunction (1, 2). As coordinated autophagy and mitochondrial function have been shown to be important drivers of physiologic EC function (7–11), we sought to find a unifying pathogenetic process that explains these findings. The endosome-lysosome system plays a key role in the trafficking, recycling, and degradation of macromolecules and cell organelles, such as mitochondria. The GTPase RAB7 is crucial for regulating endosome-lysosome function, autophagy, and cell function. Hence, we hypothesized that endothelial RAB7 deficiency is a cause of endothelial dysfunction, lung vascular remodeling, and PAH.

The main findings of our study are that (a) PAECs from patients with PAH had reduced RAB7 expression and impaired endosome-lysosome function; (b) endothelial haploinsufficiency of *RAB7* induced spontaneous PH; (c) silencing of *RAB7* in human PAECs impaired endosome and lysosome function, autophagy, and angiogenesis and promoted cellular senescence; (d) RAB7 insufficiency reduced mitochondrial membrane potential ΔΨ_m_ and oxidative phosphorylation, while promoting mitochondrial ROS production and glycolysis; (e) the RAB7 GTPase activator ML-098 reduced severe PH and right-heart dysfunction induced by chronic Hx/Su.

Whereas all layers of the PA wall and all mural cell types are affected, EC dysfunction has emerged as one of the key drivers of PA remodeling in PAH (27). ECs not only provide an important barrier, but in the pulmonary circulation they also affect smooth muscle cell function by regulation of, e.g., vasotonus or smooth muscle cell growth through the release of mediators (27). In addition, altered EC function may contribute to lung vascular remodeling by microvascular dropout following EC apoptosis and to the development of complex plexiform lesions through aberrant cell growth (4, 28–30). Yet current therapeutic avenues are unsuccessful in restoring physiologic EC function and reversing PA remodeling.

Endosomes develop as plasma membrane invaginations during endocytosis and are a shuttle for macromolecules from the outside of the cell into the cell (31). In addition, the internalization of receptors and other macromolecules from the cell membrane also occurs through the endosome system. After initial formation, endosomes are designated “early endosomes” and carry specific surface molecules, including early endosome antigen 1 (EEA1) and members of the RAB family of small GTPases, such as RAB5 (31, 32). During sorting, endosomal content, including macromolecules and cell organelles, is either recycled to the cell surface or designated for degradation via autophagy. Upon maturation to multivesicular “late endosomes,” these fuse with lysosomes to endolysosomes as sites of autophagy (31). RAB7 is a GTPase with a central function in endosomal sorting, trafficking, and fusion with lysosomes (31). Not surprisingly, loss of RAB7 impairs autophagy and particularly autophagy of mitochondria, or mitophagy, and hence cell function (15, 18–20, 33–37). Our results show that PAECs, but not PASMCs, from patients with PAH had reduced expression of RAB7 and impaired endosomal trafficking and/or degradation of dextran. Consequently, endothelial *RAB7* haploinsufficient mice developed spontaneous PH under normoxic conditions and more severe PH following exposure to Hx/Su, suggesting a fundamental protective role for physiologic levels of RAB7 in PAECs. Our results further show that *RAB7* silencing in PAECs impaired endosomal and lysosomal trafficking and/or degradation, indicating that RAB7 was essential for autophagy. Although KO of the autophagy component microtubule-associated proteins 1A/1B light chain 3B (LC3B) promoted PH in a mouse model and supports our findings, some studies have hinted at a potential role for excessive autophagy in EC dysfunction and PH progression (12, 13, 38, 39). A more detailed analysis of endosome/lysosome function and degradation processes in ECs and other vascular cell types will likely be required to reveal a definitive answer.

Our focus here was on elucidating the functional role of RAB7 in PAECs, and evidence is accumulating that mitochondrial dysfunction has a central role in regulating EC dysfunction and PA remodeling (7, 9, 10, 40). RAB7 is a known modulator of mitochondrial health through mitochondrial fission and mitophagy, and we found that knockdown of *RAB7* reduced mitochondrial membrane potential ΔΨ_m_ and impaired oxidative phosphorylation. These findings are consistent with enhanced glycolysis and lactate production, as well as impaired clearance of mitochondrial ROS. Impaired mitochondrial respiration and a shift toward glycolysis are all known features of PAECs from patients with PAH and are consistent with dysregulation of EC function akin to a “Warburg effect” (7, 9, 10). An increase in mitochondrial ROS has been previously shown in bone morphogenic protein receptor 2–KO (BMPR2-KO) ECs, and a reduction of ΔΨ_m_ was caused in PAH PAECs by hypoxia-reoxygenation but not by normoxia exposure (9). Hence, our findings corroborate the notion that RAB7 deficiency contributes to impaired mitochondrial health and function in PAH PAECs.

Our results further show that silencing of RAB7 impaired angiogenesis and migration in PAECs and promoted senescence in PAECs. Impaired angiogenesis has previously been shown in PAECs and late outgrowth endothelial progenitor cells from patients with PAH (41, 42). While these studies focused on the proliferative phenotype of PAH PAECs, more recent studies have demonstrated that endothelial senescence is also present in PAH PAECs and contributes to endothelial dysfunction and the progressive nature of PA remodeling (5, 6). Our findings further show that *RAB7* knockdown affected the transcription of gene clusters affecting organ and tissue survival, cell death, cell growth, and inflammatory, developmental, and immune responses. The angiogenic deficit and senescence were also revealed in the transcriptomic profile of *RAB7*-silenced PAECs. Because of its fundamental function in endosome and lysosome function and autophagy/mitophagy, we show in our work that endothelial RAB7 had an important protective function in the pulmonary vasculature. One additional important aspect of endothelial dysfunction in PAH is EnMT (43–45). *RAB7* knockdown in vitro promoted the transcriptomic activation of pathways for fibrotic remodeling and EnMT. Our findings suggest that, while we were able to detect a consistent downregulation of the endothelial marker PECAM1 in vitro, this decrease was not accompanied by upregulation of smooth muscle cell–specific genes or the EnMT transcription factor SNAIL. In contrast, *RAB7* knockdown can promote the upregulation of SNAIL in human vascular endothelial cells (HUVECs) (46). We found in vivo that endothelial RAB7 deficiency only exaggerated PA endothelial SNAIL expression in Hx/Su-exposed, but not in normoxia-treated, mice. This suggests that the regulation of SNAIL in the context of pulmonary arterial endothelial RAB7 deficiency cannot be modeled in monocultures.

We also found that endothelial *RAB7* haploinsufficiency impaired RV capillary density, further confirming the important role of RAB7 in vascular integrity. Yet little is known about the regulation of RAB7 expression in lung ECs. In other organs and cancer cells, multiple transcription factors have been shown to regulate RAB7 transcription, including Forkhead box protein O1 (FoxO1), c-myc, and, under hypoxia exposure, STAT3 (47–49). Among these, hypoxia and STAT3 signaling are enhanced in ECs from patients with PAH (7) and repress RAB7 expression in ovarian cancer cells (48), suggesting that hypoxia and STAT3 signaling may contribute to RAB7 deficiency in ECs from patients with PAH.

To determine whether enhancing the activity of remaining RAB7 provides a therapeutic avenue, we used the GTPase activator ML-098, which has a low median EC_50_ for RAB7 and a much weaker affinity for other small GTPases (50). To our knowledge, our study was the first to use ML-098 in in vivo experiments. According to our findings, ML-098 treatment not only prevented but also reduced established PH in rats exposed to Hx/Su. ML-098 has only been used in cell culture experiments, and studies have shown protection from age-related deterioration of oocytes (19), consistent with our observation that loss of RAB7 promoted senescence.

Our study has some limitations. First, our work focused on the role of RAB7 in EC function and lung vascular remodeling, but other aspects of RAB7 deficiency, such as an exaggerated inflammatory response, may also be mechanistically relevant. Second, we tested ML-098 only in the Hx/Su model of PH, although this is one of the most relevant models mimicking many features of PAH, including the progressive nature of the condition and the occlusive pulmonary arteriopathy (22, 51).

Taken together, we demonstrated in PAECs a fundamental role for the endolysosomal GTPase RAB7 in physiologic cell function, transcriptomic profile, endolysosome function, mitochondrial health, and pulmonary vascular integrity. Pharmacologic modulation of RAB7 function could complement existing therapeutic strategies and offer a new therapeutic avenue for patients with PAH.

## Methods

### Reagents and constructs

#### Cell lines.

Primary human PAECs and PASMCs from male and female patients with PAH and control patients (failed donors, no known pulmonary vascular disease) were obtained from the Pulmonary Hypertension Breakthrough Initiative (PHBI) and Cell Processing Cores at the University of Pittsburgh and the University of California Davis. The use of deidentified human primary cell lines was deemed “nonhuman subjects research” by the Office of Research Subjects Protection at OSU.

#### Tissue samples.

Deidentified formalin-fixed, paraffin-embedded 5 μm tissue sections were obtained from the PHBI. The use of deidentified human tissue samples was deemed “nonhuman subjects research” by the Office of Research Subjects Protection at OSU.

#### Animal models.

*RAB7^fl/WT^* Cdh5-Cre mice were generated by crossbreeding *RAB7^fl/fl^* mice (52) [strain B6.129(Cg)-*Rab7^tm1.1Ale^*/J, no. 021589, The Jackson Laboratory] with *Cdh5*-Cre mice (53) [strain B6.Cg-Tg(*Cdh5*-Cre)1Spe/J, no. 033055, The Jackson Laboratory]. *RAB7^fl/fl^* mice and *Cdh5*-Cre mice are on a C57BL/6J background. The mice used in the experiments were crossbred for at least 5 generations. Male and female mice aged 8–16 weeks were used for the experiments. Genotyping was performed by Transnetyx. Littermate *RAB7^fl/WT^* Cdh5-Cre^–^ mice were used as controls in the experiments. For rat experiments, male Sprague-Dawley rats (Hsd:Sprague Dawley SD) were obtained from Inotiv at the age of 6 weeks.

### Animal experiments

For the Hx/Su rat model, 6-week-old male rats were treated s.c. with SU5416 (20 mg/kg body weight, MilliporeSigma, S8442) at the beginning of 3 weeks of chronic hypoxia exposure (inspiratory oxygen fraction, 10%) in a normobaric nitrogen dilution chamber (Biospherix), as published previously (54). For the Hx/Su mouse model, mice were given 20 mg/kg SU5416 s.c. once a week during a 3-week exposure to chronic hypoxia (54). ML-098 (TargetMol, T4619) was diluted in DMSO and given to male rats after dilution in 0.9% saline (final 1% DMSO) at 0.1, 1.0, and 10 mg/kg body weight, 5 times a week from day 1 to day 21 (preventive) during the chronic hypoxia phase of the Hx/Su model or after return to normoxia from day 22 to day 35 or day 36 to day 49. For all treatments, animals were randomly assigned to the treatment groups, and treatments were given in a blinded manner. At the indicated time points, echocardiography was performed using a Vevo 2100 system (VisualSonics) located at the Small Animal Imaging Facility at OSU or a GE Vivid IQ Premium system (GE Healthcare) under isoflurane anesthesia (rats) or under ketamine/xylazine anesthesia (mice). Echocardiographic estimation of the cardiac output was calculated according to a previous report (55). Terminal right-heart catheterization was done with a 1.4F Millar catheter and a PowerLab acquisition system (AD Instruments). For this procedure, the animals were anesthetized with ketamine/xylazine and ventilated after tracheostomy with a small animal ventilator (RoVent, Kent Scientific Corporation). A median sternotomy was performed to open the chest cavity, and the 1.4F Millar catheter was inserted following a small puncture of the right ventricle. Right ventricular hemodynamics were recorded over at least 5 minutes for steady-state measurements. The acquisition and analysis of echocardiographic and hemodynamic data were done in a blinded manner using numerical coding.

### Cell culture experiments

Human PAECs were cultured in complete endothelial growth medium-2 microvascular (EGM-2MV) (Lonza, CC-3162) in a cell culture incubator at 37°C with 5% CO_2_ and 100% humidity. Human PASMCs were cultured in complete smooth muscle cell growth medium 2 (SmGM-2) (Lonza, CC-3182). For siRNA-mediated knockdown of RAB7, PAECs were transfected with 50 nM *RAB7* or control siRNA using GenMute Reagent (SignaGen, SL100568). The following siRNAs were used: *RAB7* siRNA (Integrated DNA Technologies [IDT], NM_004637 13.2) and control siRNA (IDT, 51-01-14-04). After 24 hours, siRNA was removed, and experiments were performed 48 or 72 hours after transfection. To test 2D angiogenic network formation, cells were seeded on Matrigel in a μ-slide 15-well (Ibidi, 81506), and images of network formation were acquired at ×4′ magnification using an EVOS M7000 (Thermo Fisher Scientific, AMF7000) automatic microscope. The images were analyzed using AngioTool software (NCI). To test gap closure, ECs were seeded in 3-well migration assay culture inserts (Ibidi, 80369). Removal of the insert left a well-defined uniform gap, and gap closure was observed after 16 hours as described previously (54). To detect cellular senescence, cells were stained with SA–β-gal according to the manufacturer’s instructions for the Senescence β-Galactosidase Staining Kit (Cell Signaling Technology, 9860). Cells were counterstained with DAPI. SA–β-gal activity was determined by the detection of blue-green precipitate over the cells. Cells were viewed using an EVOS M7000 automated fluorescence microscope (Invitrogen, Thermo Fisher Scientific) under bright-field illumination. To evaluate vesicular trafficking and degradation, cells were incubated with pHrodo Red dextran (Invitrogen, Thermo Fisher Scientific, P10361) for 20 minutes, followed by fixation with 10% formalin. To specifically determine endosomal localization of dextran, GFP-conjugated RAB5A (early endosome marker) was expressed via a BacMam 2.0 baculovirus construct using the CellLight Early Endosomes-GFP kit (Thermo Fisher Scientific, C10586). To obtain the lysosomal localization of dextran, lysosomes were stained with LysoTracker Green (Thermo Fisher Scientific, L7526). To measure lysosome activity, a cathepsin B activity assay was performed according to the manufacturer’s recommendations (Cathepsin B Assay Kit, PromoCell, PK-CA577-K140). To test mitochondrial membrane potential, the TMRE staining kit was used (200 nM, Abcam, ab113852), combined with MitoTracker Green (MitoGreen, 100 nM, Thermo Fisher Scientific, M7514) staining. Staining was performed in FCS-free media for 30 minutes. Carbonyl cyanide *p*-trifluoro methoxyphenylhydrazone (FCCP) (included in the TMRE staining kit) was added 10 minutes before staining at 20 μM. Analysis was performed using a BD FACSSymphony A1 flow cytometer and FlowJo 10 software. Data are presented as fractions of TMRE^hi^ MitoGreen^hi^ (physiologic ΔΨm) and TMRE^lo^ MitoGreen^hi^ cells (decreased ΔΨ_m_). ΔΨ_m_ distribution was also determined using TMRM staining (TMRM Assay Kit, Abcam, ab228569) on adherent PAECs followed by nuclear staining with Hoechst 33342 (Thermo Fisher Scientific, H-1399). To determine mitochondrial ROS production, cells were stained with 2 μM mitoSOX mitochondrial ROS stain (mitoSOX Red, Thermo Fisher Scientific, M36008) for 20 minutes, combined with the LIVE/DEAD Fixable Near-IR Dead Cell Staining Kit (Thermo Fisher Scientific, L34975), followed by analysis on a BD FACSSymphony A1 flow cytometer with FlowJo 10 software. Lactate production was measured with the Lactate-Glo Assay (Promega, J5021) according to the manufacturer’s recommendations.

Details regarding the determination of mitochondrial dynamics, Seahorse OCR/ECAR measurements, protein isolation, immunoblotting, RNA isolation, bulk RNA-Seq, histology, immunohistochemistry, and microscopy are found in the Supplemental Methods.

### Statistics

The normal distribution of data was tested with the D’Agostino-Pearson or Shapiro-Wilk test. Normally distributed data are shown as single data points and the mean ± SD and were compared using a 2-tailed Student’s *t* test (2 groups) or 1- or 2-way ANOVA (more than 2 groups), followed by multiple-comparison corrections using the Holm-Šidák or Šidák test. Data that were not normally distributed are presented as the median ± IQR and were compared using a nonparametric 2-tailed Mann-Whitney *U* test (2 groups) or a Kruskal-Wallis test (more than 2 groups), followed by Dunn’s multiple-comparison test. When 2 variables were analyzed, a 2-way ANOVA was used if the residual distribution was normally distributed. If residuals were not normally distributed, a Kruskal-Wallis analysis was used to compare the outcome distribution between the groups. The calculations were performed using GraphPad Prism 9.0 (GraphPad Software). A *P* value of less than 0.05 was considered significant. Data are presented as the mean ± SD, median ± IQR, or geometric mean, as indicated in the Figure legends.

### Study approval

The use of deidentified human tissue samples and cell lines was deemed “nonhuman subjects research” by the Office of Research Subjects Protection at OSU. PAECs and PASMCs were isolated in a deidentified manner under PHBI-approved protocols with institutional ethics board approval in accordance with the revised ethics guidelines of the Declaration of Helsinki of 1983. Informed consent was waived by these IRBs. Animal experiments were approved by the IACUC of OSU under protocol no. 2019A00000092.

### Data availability

Values for all data points in graphs are reported in the Supplemental Supporting Data Values file. Original uncropped Western blots are included in the Supplemental Data file. Raw data files from bulk RNA-Seq data have been deposited in the NCBI’s Gene Expression Omnibus (GEO) database (GEO GSE243774). Data can also be obtained from the corresponding author upon request.

## Author contributions

BP, SB, NS, JCH, ARB, HY, MR, ALM, RKM, EAG, DME, and LF conceived and designed the study. BP, SB, TH, DF, LR, ACG, GN, JAOR, ARB, TVK, PY, DME, and LF performed experiments and obtained and analyzed data. BP, SB, DME, and LF wrote the initial draft of the manuscript. All authors contributed to the revision of the manuscript and approved the final version. The order of the authors’ names was determined on the basis of their overall contribution to data acquisition and manuscript preparation.

## Supplementary Material

Supplemental data

Unedited blot and gel images

Supporting data values

## Version 1 11/28/2023

In-Press Preview

## Version 2 02/01/2024

Electronic publication
