# Supplementary material for: RAB7 deficiency impairs pulmonary artery endothelial function and promotes pulmonary hypertension
Source: J Clin Invest. 2024 Feb 1;134(3):e169441. doi: 10.1172/JCI169441 (PMC10836802; doi:10.1172/JCI169441)
Supplement: Supplemental data [file jci-134-169441-s102.pdf]

Supplemental Material

RAB7 deficiency impairs pulmonary artery endothelial function and promotes pulmonary hypertension.

Bryce Piper, Srimathi Bogamuwa, Tanvir Hossain, Daniela Farkas, Lorena Rosas, Adam Green, Geoffrey Newcomb, Nuo Sun, Jose A. Ovando, Jeffrey C. Horowitz, Aneel R. Bhagwani, Hu Yang, Tatiana V. Kudryashova, Mauricio Rojas, Ana L. Mora, Pearlly Yan, Rama K. Mallampalli, Elena A. Goncharova, David M. Eckmann, Laszlo Farkas

Supplemental Methods

Determination of Mitochondrial Dynamics

Mitochondrial motility (distribution of net mitochondrial movement) was determined using wide-field fluorescence microscopy and image analysis as described in significant detail in our previous work (1). Briefly, the day prior to experiments, cells were transfected with CellLight Mitochondria-GFP, BacMam 2.0 (ThermoFisher, C10600) at 40 particles/cell and kept under dark conditions at 37 °C. Cells were also stained with DAPI and were placed in Recording HBSS (HBSS pH 7.4 with 1.3 mM $CaCl_2$, 0.9 mM $MgCl_2$, 2 mM glutamine, 0.1 g/L heparin, 5.6 mM glucose, and 1% FBS) for microscopy imaging. For image acquisition, cells were imaged using an Olympus IX-51 inverted epifluorescence microscope with an Olympus 40x oil immersion objective lens and a Hamamatsu ORCA camera (2048 X 2048 pixels) with an LED light source from Lumencor. Images were collected using Metamorph 7.10.5 software. Images of cells were captured every 3 seconds over a total duration of 5 minutes, providing 101 sequential image frames, referred to as "whole cell" frames. Image pre-processing followed by analysis of intracellular mitochondrial motility in the cell perinuclear and cell peripheral regions was performed using ImageJ and MATLAB with established methodology (2). This degree of analysis was performed to determine if intracellular inhomogeneities of mitochondrial motility were present and to quantify the measurable effects of specific cellular conditions.

Seahorse OCR/ECAR measurement

Cellular mitochondrial oxygen consumption rate (OCR) in pmol/min and extracellular acidification rates (ECAR) in mpH/min were evaluated using the Seahorse Xfe24 Analyzer (Agilent). Experiments were performed with unbuffered DMEM XF assay media (Agilent, 103575-100) supplemented with 2 mM GlutaMAX, 1 mM sodium pyruvate, and 5 mM glucose (OCR experiments only) at pH 7.4 and 37 °C. Following instrument calibration, select compounds were injected to obtain basal respiration, leak, and maximum respiration (Max) in OCR experiments. After initial measurement of basal respiration, oligomycin (2 μM) was injected to inhibit ATP synthase (Complex V) to obtain LEAK. FCCP (2.5 μM) was subsequently injected to induce uncoupling that yields Max. We also obtained spare respiratory capacity, which assesses mitochondrial reserve for ATP production in response to increased metabolic demand. Spare respiratory capacity (SRC) is equal to the difference between maximal and basal respiration. In the ECAR experiments, sequential administration of glucose (10 mM) and oligomycin (1 μM) enables measurement of glycolysis and glycolytic capacity, respectively. Subsequent injection of 2-deoxy glucose (50 mM) enables calculation of glycolytic reserve and non-glycolytic acidification.

These respiratory states and parameters of glycolytic function were measured at consecutive time points, which were averaged to give a single value for each state for each cell culture well. Data were normalized using metabolic activity; background readings for each plate were calculated by averaging the OCRs/ECARs from the background wells for those plates and were subtracted from all subsequent readings. Erratic or extreme background well readings were excluded. All experiments were

repeated in duplicate runs performed on separate days. Each well was analyzed individually, and the resultant data are presented as the mean ± standard deviation (SD) for all experiments.

Protein isolation and immunoblotting

Protein isolation and Western blotting were performed as published previously (3-5). Following lysis with RIPA buffer with proteinase and phosphatase inhibitors, 10-20 µg protein (cell lysate) and 40 µg protein (whole lung lysate) were resolved by SDS-PAGE at 90 V for 60-80 min. Then, the protein was blotted at 270 mA for 2 hours onto a nitrocellulose membrane for antibody staining and chemiluminescence detection (ECL). Membranes were blocked with 5% bovine serum albumin/0.5% Tween20/TBS for 1 hour. Primary antibody incubation was performed in 5% bovine serum albumin/0.5% Tween20/TBS overnight at 4 °C, followed by secondary antibody incubation in 5% bovine serum albumin/0.5% Tween20/TBS for 1h (horse radish peroxidase-conjugated secondary antibodies). Images were obtained by developing an ECL solution and image acquisition with a Biorad ChemiDoc gel imager with ImageLab software (Biorad, Hercules, CA). Densitometry with automated background subtraction was performed with ImageLab (Biorad, Hercules, CA). The following primary antibodies were used: α-SMA (DAKO, M0851), α-tubulin (Proteintech, 66031), β-actin (Millipore Sigma, A5441), CALPONIN 1 (Cell Signaling, 17819), p16 (BD Biosciences, 551153), PECAM1 (Cell Signaling, 3528), RAB7 (Abcam, ab137029), RAB7 (Cell Signaling, 957465), SM-22α (Cell Signaling, 52011), SNAIL (Cell Signaling, 3879), VE-cadherin (Novus Biologicals, NB110-60978), vWF (Agilent, A0082).

RNA sequencing and analysis

RNA isolation was performed as published previously (3-5). RNA was isolated using the Qiagen miRNAeasy mini kit (217084) according to the manufacturer's instructions, followed by DNAse digestion to remove potential genomic DNA contamination. DNAse I treatment was performed at 0.1 U/µl final activity for 15 min at room temperature, followed by the addition of 25 mM EDTA and incubation at 65˚C for 10 min to inactivate DNAse. Total mRNA concentrations were measured using Nanodrop. RNA sequencing was performed in the Genomic Shared Resource at The Ohio State University using NEBNext® Ultra^{TM} II Directional RNA Library Prep Kit (New England Biolabs, E7760) for Illumina, NEBNext rRNA Depletion Kit (New England Biolabs, E6310), and NEBNext Multiplex Oligos for Illumina Unique Dual Index Primer Pairs (New England Biolabs, E6440). 100 ng total RNA (quantified using a Qubit Fluorometer) was used as library input. Fragmentation time was 10 min, and PCR was done 11x. Libraries were sequenced with Novaseq SP Pair_end 150 bp format.

Raw Data was analyzed by ROSALIND® (https://rosalind.bio/), with a HyperScale architecture developed by ROSALIND, Inc. (San Diego, CA). Reads were trimmed using cutadapt (6). Quality scores were assessed using FastQC (7). Reads were aligned to the Homo sapiens genome build GRCh38 using STAR (8). Individual sample reads were quantified using Htseq (9) and normalized via Relative Log Expression (RLE) using DESeq2 R library (10). Read Distribution percentages, violin plots, identity heatmaps, and sample MDS plots were generated as part of the QC step using RseQC (11). Deseq2 was also used to calculate fold changes and p-values and perform optional covariate

correction. The clustering of genes for the final heatmap of differentially expressed genes was done using the PAM (Partitioning Around Medoids) method using the fpc R library (12). Hypergeometric distribution was used to analyze the enrichment of pathways, gene ontology, domain structure, and other ontologies. The topGO R library (13) was used to determine local similarities and dependencies between GO terms in order to perform Elim pruning correction. Several database sources were referenced for enrichment analysis, including Interpro (14), NCBI (15), MsigDB (16, 17), REACTOME (18), and WikiPathways (19). Enrichment was calculated relative to a set of background genes relevant for the experiment. Following the identification of differentially expressed genes with fold changes of >1.25 and an adjusted P value of <0.05, downstream analysis of functions and pathway enrichment was performed using Ingenuity Pathway analysis (Qiagen). Clustered heatmaps were generated with Rosalind, and a summary of functions was assembled in GraphPad Prism 9.0.

Histology and immunohistochemistry

Tissue processing and Masson Trichrome staining were done by Histowiz. Immunohistochemistry and double immunofluorescence stainings were performed according to previous publications (3, 5). In brief, for immunohistochemistry, rehydration of slides was followed by antigen retrieval (20 min. heating in boiling citrate buffer at pH 6.0 in most protocols, except for vWF, which used proteinase K 1:50 for 5 min as antigen retrieval). Blocking of endogenous peroxidase (H_2O_2) and of unspecific binding with 1% normal swine serum (NSS)/PBS for 15 min was followed by incubation with primary antibodies (diluted in 1% NSS/PBS) overnight at 4°C. Further steps were incubation with

secondary biotin-labeled antibody (1 hour, diluted in 1% NSS/PBS) and incubation with HRP conjugated Streptavidin for 1 hour. Slides were stained with diaminobenzidine solution and counterstained with Mayer's hematoxylin. After dehydration, slides were mounted with a coverslip and permanent mounting medium. The following antibodies were used for immunohistochemistry: α-SMA (DAKO, M0851), vWF (Millipore Sigma, A0082), and PCNA (Cell Signaling, 25865).

Immunofluorescence required rehydration followed by antigen retrieval (20 min. heating in boiling citrate buffer pH 6.0). After blocking unspecific binding with 1% normal swine serum (NSS)/PBS for 15 min, primary antibody #1 was added 1% NSS/PBS for overnight incubation at 4°C. Incubation with secondary fluorescence-labeled antibody (4 hours, diluted in 1% NSS/PBS) was followed by incubation with primary antibody #2 overnight at 4°C and 4h incubation with secondary fluorescence-labeled antibody #2 for 4h, followed by nuclear staining with DAPI for 5 min. Then, slides were mounted with coverslip and SlowFade Gold (ThermoFisher, S36936). The following antibodies were used: α-SMA (DAKO, M0851), RAB7 (Abcam, ab13029), SNAIL (Invitrogen, PA5-119607), vWF (Millipore, MAB3442), vWF (Agilent, A0082). For EC detection, we further used Texas-Red-conjugated Tomatolectin (*Lycopersicon esculentum*, LEL, Vector Labs, TL-1176-1) following antigen retrieval with overnight incubation at 4°C. The following secondary antibodies were used: AlexaFluor® 488-labeled anti-mouse IgG1 (LifeTechnologies, A21121), AlexaFluor® 647-labeled anti-mouse IgG_{2a} (LifeTechnologies, A21241), AlexaFluor® 488-labeled anti-rabbit (LifeTechnologies, A11008), and AlexaFluor® 647-labeled anti-rabbit (LifeTechnologies, A21244).

Media wall thickness (MWT) was analyzed as described previously (3, 4). In brief, images of pulmonary arteries were acquired with an EVOS M7000 automated microscope. Treatment groups were masked by numerical coding. Media thickness (MT) and external diameter (ED) were measured and MWT was calculated as MWT=[(2×MT)/ED]×100%. Pulmonary arteries were categorized as small-sized 25 μm < ED < 50 m and medium-sized 50 μm ≤ ED < 100 μm. For each animal, 30-40 pulmonary arteries were measured in two orthogonal directions using Fiji image analysis software. Each MWT data point represents the average of all analyzed pulmonary arteries of one animal. Pulmonary artery occlusion in rats was analyzed as published previously by us using slides stained for vWF immunohistochemistry and identifying the fraction of small-sized pulmonary arteries that were classified as 'patent', 'partially occluded' and 'completely obstructed' (3, 5). Each occlusion data point represents the average of pulmonary arteries with no, partial, and full occlusion of all analyzed pulmonary arteries of one animal.

For analysis of PCNA$^+$ cells per pulmonary artery, images of pulmonary arteries were randomly acquired from transversal sections stained for PCNA immunohistochemistry at 40× magnification. The number of PCNA$^+$ cells was divided by the number of nuclei/pulmonary artery wall cross-section to calculate the fraction of PCNA$^+$ cells. Each PCNA data point represents the average of all analyzed pulmonary arteries of one animal.

For quantification of α-SMA$^+$ ECs and SNAIL$^+$ ECs, a minimum of 10 pulmonary arteries was acquired by confocal microscopy, and the number of α-SMA$^+$ ECs and

SNAIL$^+$ ECs and the total number of ECs per pulmonary artery were quantified. Results were expressed as the average per animal of the fraction of positive ECs vs. total ECs.

To quantify fluorescence intensity, the EC marker vWF was used to detect ECs and intima cells. The MFI of RAB7 staining was measured using ImageJ. Background MFI (area without positive staining) was subtracted. The fluorescence intensity was then normalized to the average of the control samples.

To determine the capillary density in the RV, fluorescent images (400× magnification) were acquired of the LEL-stained RV, the capillary area (LEL$^+$ area) was measured, and capillary density was calculated as a fraction of LEL$^+$ area vs. total tissue area (obtained from background fluorescence). To obtain the RV cardiomyocyte cross-sectional area, images were obtained from whole tissue scanning of Masson Trichrome stained sections as done by Histowiz. The crossectional area of cardiomyocytes was measured using ImageJ in randomly obtained images. All values from one animal were averaged and presented as RV cardiomyocyte crossectional area for each animal.

For all histomorphometric analyses, objectivity was ensured by masking treatment groups through numerical coding and an investigator unaware of the treatment groups.

Microscopy

Light microscopy and wide-field fluorescence images were acquired using an EVOS M7000 automated microscope (Thermo Fisher Scientific, Waltham, MA). Optical sections of immunofluorescence were acquired using an inverted Olympus FV3000 confocal microscope system located at the OSU Campus Microscopy and Imaging Facility. The images were assembled with Fiji software (20).

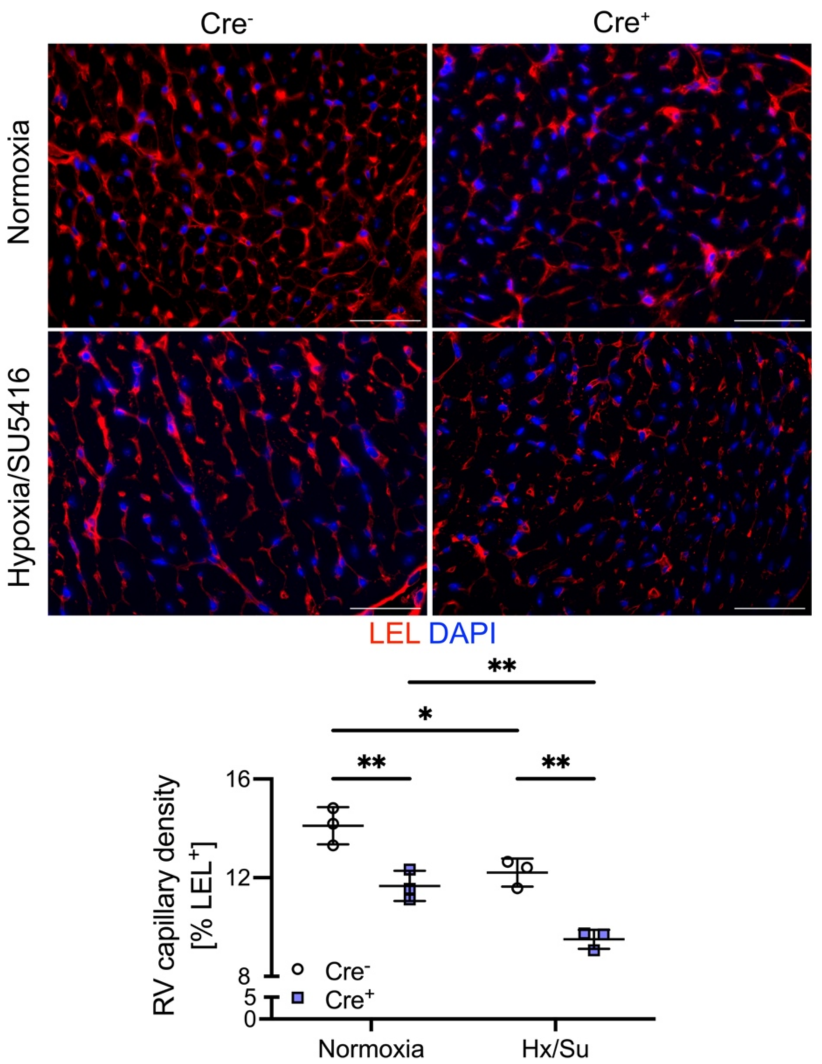

Supplemental Figure 1. Right ventricular capillary density in *RAB7*$^{fl/wt}$Cdh5-Cre mice. Representative fluorescence microscopy images of capillary density (Tomatolectin, *Lycopersicon esculentum* lectin, LEL, red pseudocolor) from the right ventricles and quantification of the fraction of LEL^{+} capillary area show impaired capillary density in *RAB7*$^{fl/wt}$Cdh5-Cre^{+} mice. Scale bars: 25 µm. n=3 per group. Nuclear staining with DAPI. Data are shown as single data points and mean ± SD. Data were analyzed by two-way ANOVA with Holm-Sidak multiple comparison test. *P<0.05, **P<0.01.

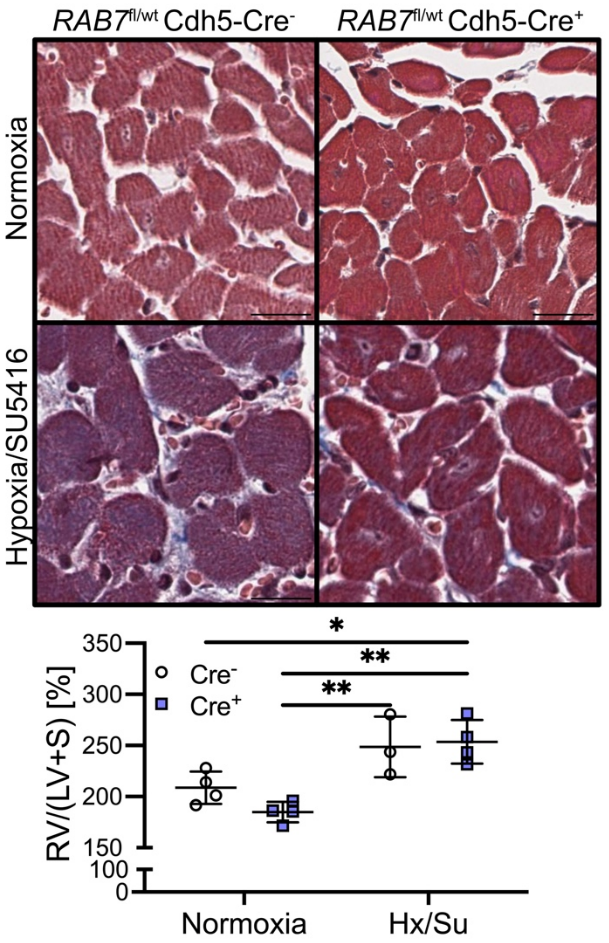

Supplemental Figure 2. Right ventricular cardiomyocyte crossectional area in *RAB7*$^{fl/wt}$Cdh5-Cre mice. Representative images of the right ventricles (Masson Trichrome staining) and quantification of the right ventricular cardiomyocyte cross-sectional area (CM CSA) show increased CM CSA in *RAB7*$^{fl/wt}$Cdh5-Cre^{-} mice and *RAB7*$^{fl/wt}$Cdh5-Cre^{+} mice exposed to chronic hypoxia/SU5416. Scale bars: 25 µm. n=3-4 per group. Data are shown as single data points and mean ± SD. Data were analyzed by two-way ANOVA with Holm-Sidak multiple comparison test. *P<0.05, **P<0.01.

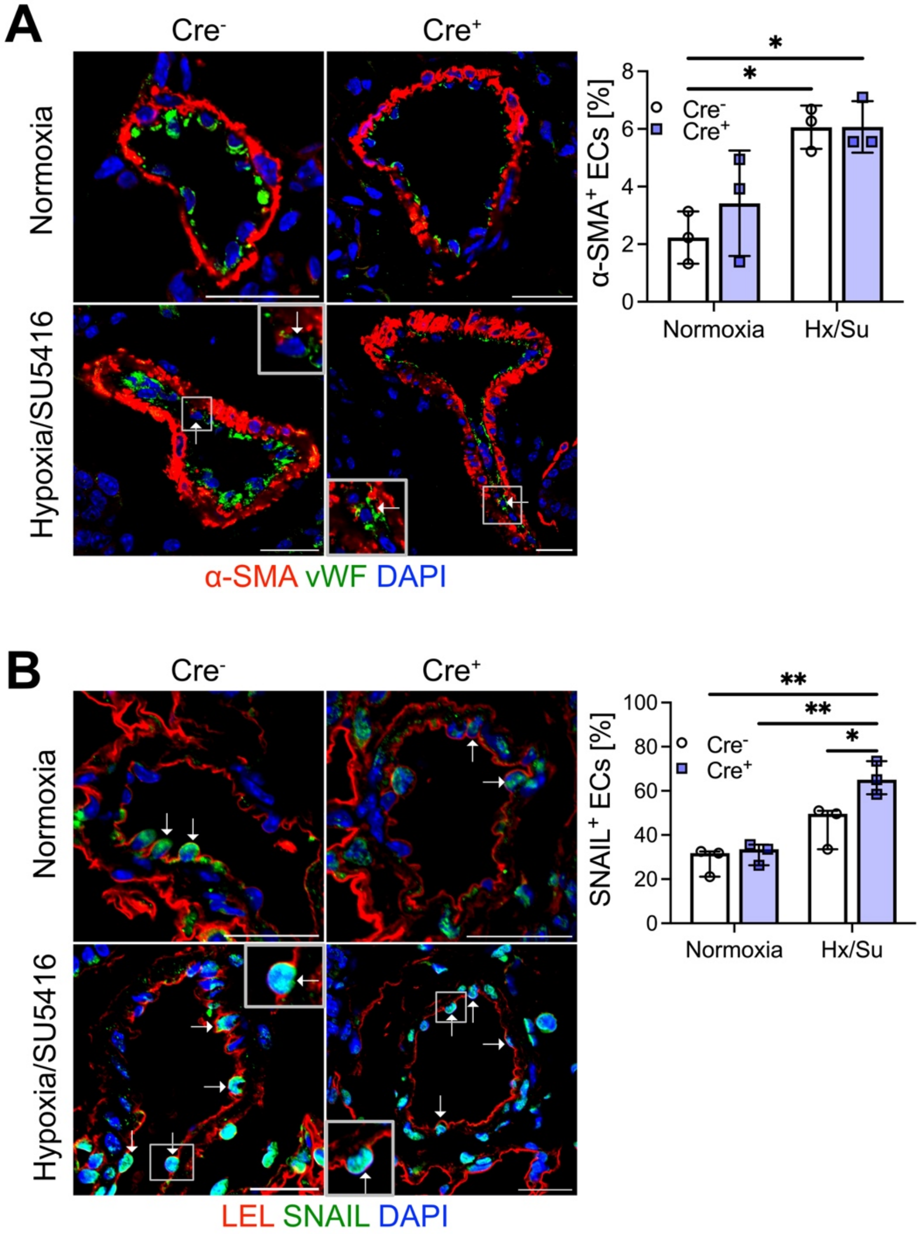

Supplemental Figure 3. In situ endothelial-to-mesenchymal transition in the mouse model of endothelial *Rab7* haploinsufficiency. (**A**) Representative optical sections (confocal microscopy) of α-smooth muscle actin (α-SMA, red pseudocolor) and vWF (green pseudocolor) staining show evidence for endothelial-to-mesenchymal transition (EnMT) by detecting cells expression endothelial (vWF) and mesenchymal (α-SMA) marker. The inserts show the cells expressing both markers identified by arrow and dotted rectangle in more detail. The graph shows the quantification of α-SMA^{+} ECs in n=3 animals per group. Scale bars: 25 µm. (**B**) Representative optical sections (confocal microscopy) of *Lycopersicon esculentum* lectin-stained cells (LEL, Tomatolectin, red pseudocolor) and SNAIL (green pseudocolor) staining show evidence for endothelial-to-mesenchymal transition by detecting cells expression endothelial cell marker (LEL) and EnMT transcription factor Snail (nuclear SNAIL staining by overlap between green and

blue pseudocolor, arrows). The inserts show the double positive cells identified by an arrow and dotted rectangle in more detail. The graph shows the quantification of Snail$^+$ ECs in n=3 animals per group. Scale bars: 25 µm. For the analysis, ≥10 pulmonary arteries were analyzed for each mouse, and the average values for all pulmonary arteries per mouse were used for statistical analysis. Nuclear staining with DAPI. The data are presented as single data points and mean ± SD (A) or median ± range (B) and were compared using two-way ANOVA followed by Holm-Sidak multiple comparison test and D'Agostino-Pearson test for normality of residuals. *P<0.05, **P<0.01, ***P<0.001.

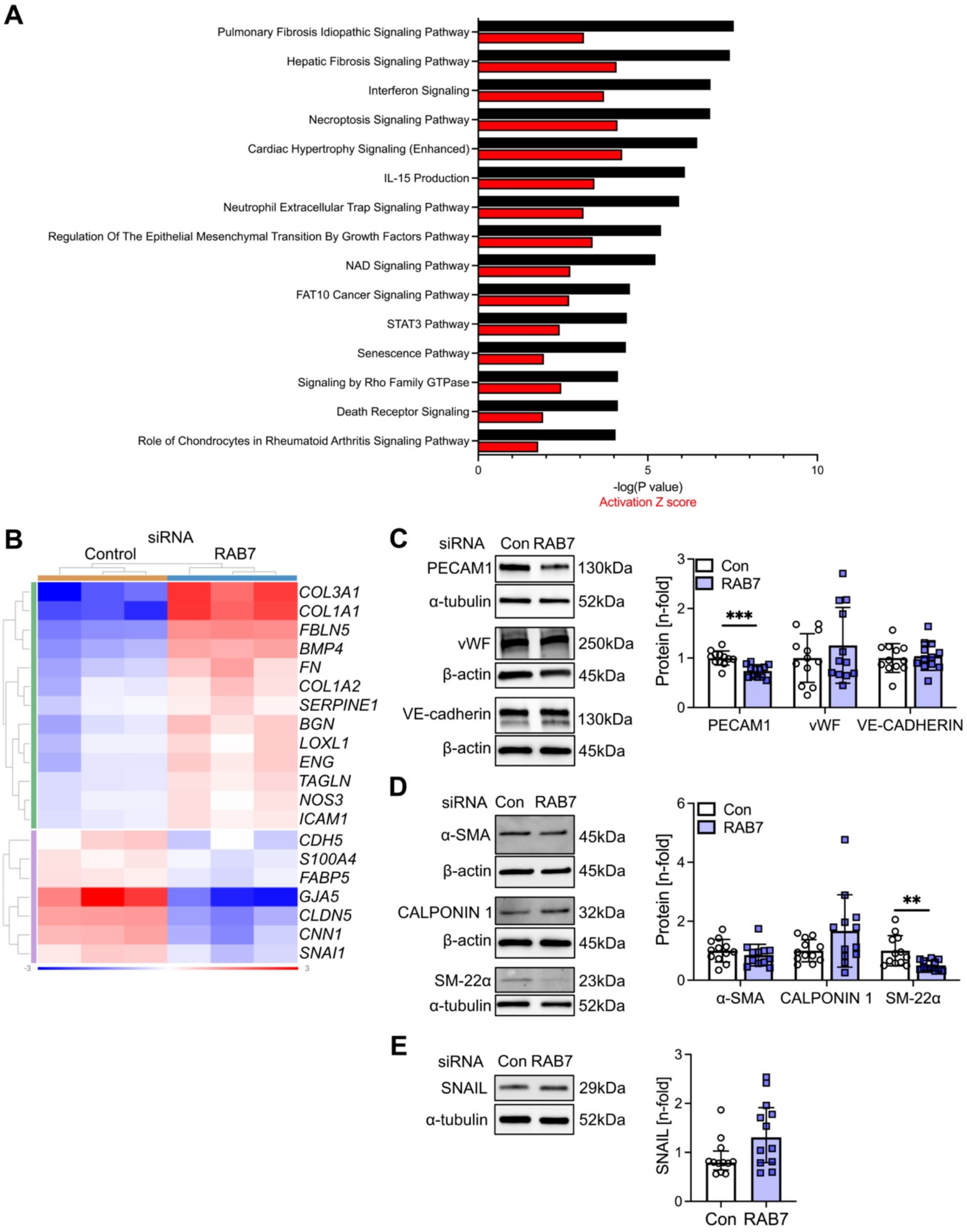

Supplemental Figure 4. In vitro analysis of endothelial-to-mesenchymal transition in pulmonary artery ECs transfected with *RAB7* or control siRNA. (**A**) Ingenuity pathway analysis of bulk RNAseq of pulmonary artery ECs (PAECs) following RAB7 knockdown. The diagram shows the 15 most activated pathways as per the highest -log(P value) and a Z activation score of ≥|1.5|. Black bars indicate -log(P value), and red bars

indicate positive (activation) Z score. DEGs were obtained from a fold-change cut-off of > |1.25| and adjusted P <0.05. (**B**) Hierarchically clustered heatmap of bulk RNAseq data of PAECs transfected with *RAB7* siRNA vs. control siRNA shows differentially expressed genes implicated in endothelial-to-mesenchymal transition (EnMT), including endothelial markers (e.g., *NOS3*, *CDH5*, *GJA5*, *CLDN5*), mesenchymal/smooth muscle cell markers (e.g., *TAGLN, CNN1*), extracellular matrix components (e.g., *COL3A1, COL1A1, FN, COL1A2)* and the EnMT transcription factor *SNAI1* (encoding SNAIL). The data are shown as $\log_2$-fold changes normalized to the row mean with a fold-change cut-off of > |1.25| and adjusted P <0.05. (**C**) Representative immunoblots and densitometric quantification indicate the downregulation of one endothelial marker, PECAM-1, following *RAB7* knockdown. (**D**) Representative immunoblots and densitometric quantification show no upregulation of mesenchymal/smooth muscle cell markers following *RAB7* knockdown. (**E**) Representative immunoblots and densitometric quantification of EnMT transcription factor SNAIL in PAECs following *RAB7* knockdown. β-actin and α-tubulin were run on the same blot and samples. Note: β-actin for VE-cadherin in (C) and calponin in (D) is identical due to using the same blot. Data in (C-D) are pooled from 3 control PAEC lines, each n=4. Data are shown as single data points and mean ± SD (C, D) or median ± interquartile rank (E) of n-fold of normalized protein expression vs. control siRNA. The data were compared using a 2-tailed Student's t-test (C, D) or a 2-tailed Mann-Whitney test (E). **P<0.01, ***P<0.001.

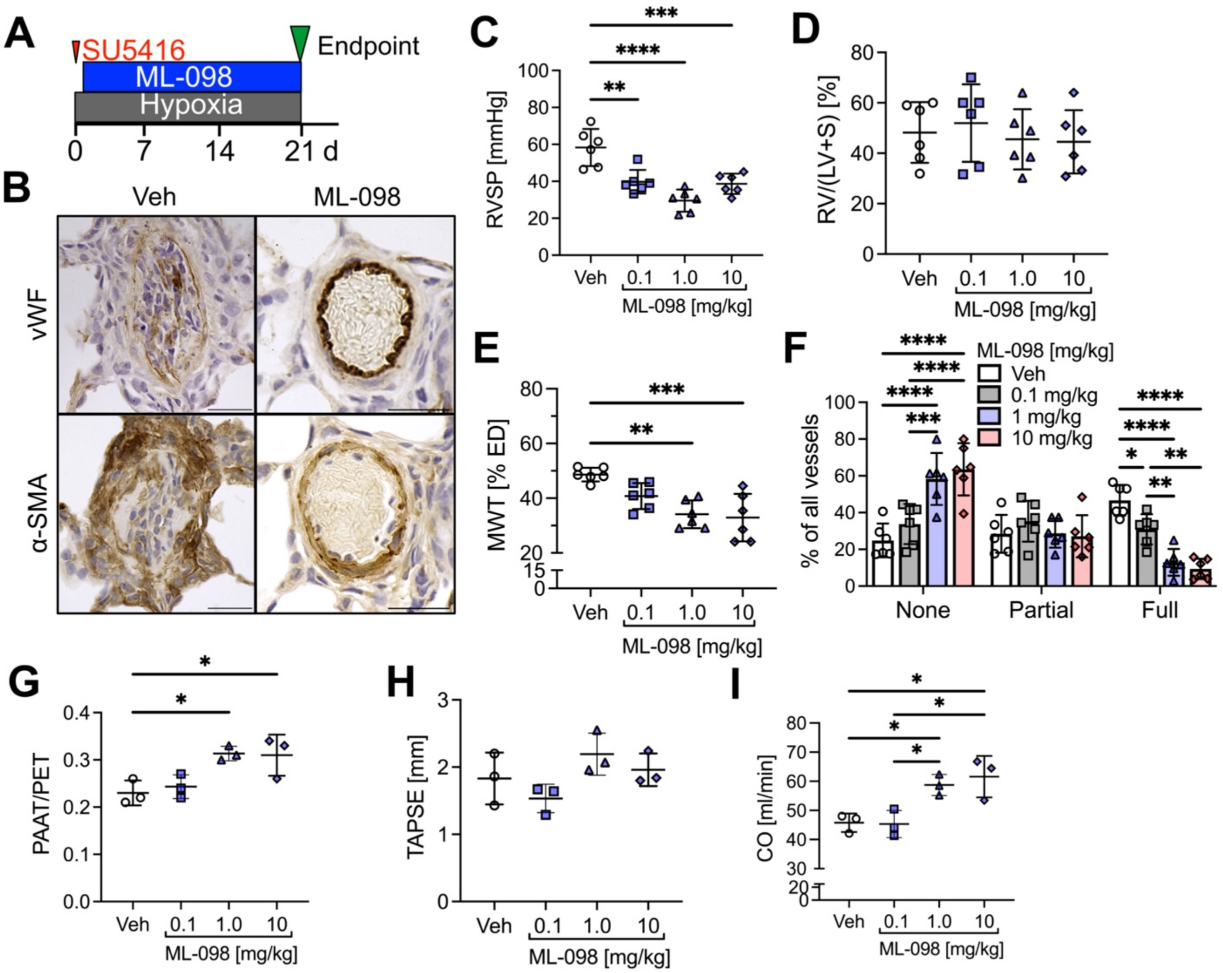

Supplemental Figure 5. Preventive dose-finding study for ML-098 in chronic hypoxia/SU5416 induced PH in rats. (**A**) Preventive treatment diagram. (**B**) Representative von Willebrand Factor (vWF) and α-smooth muscle actin (α-SMA) immunohistochemistry images (same pulmonary arteries from serial section). (**C**) right ventricular systolic pressure (RVSP), (**D**) Fulton index, (**E**) pulmonary artery media wall thickness (MWT), and (**F**) occlusion of small pulmonary arteries. (**G-I**) Echocardiographic parameters (G) ratio of pulmonary artery acceleration time (PAAT) vs. pulmonary ejection time (PET), (H) tricuspid annual plane systolic excursion (TAPSE) and (**I**) echocardiographically estimated cardiac output (CO). All graphs show single values and mean ± SD. n=6 per group (C-F), n=3 (G-**I**) (male rats). Note: echocardiography was only performed on n=3 rats per group. Data were compared using a 2-tailed Student's t-test (C-E, G-**I**) and 2-way ANOVA with Holm-Sidlak multiple comparison test (F). *P<0.05, **P<0.01, ***P<0.001, ****P<0.0001.

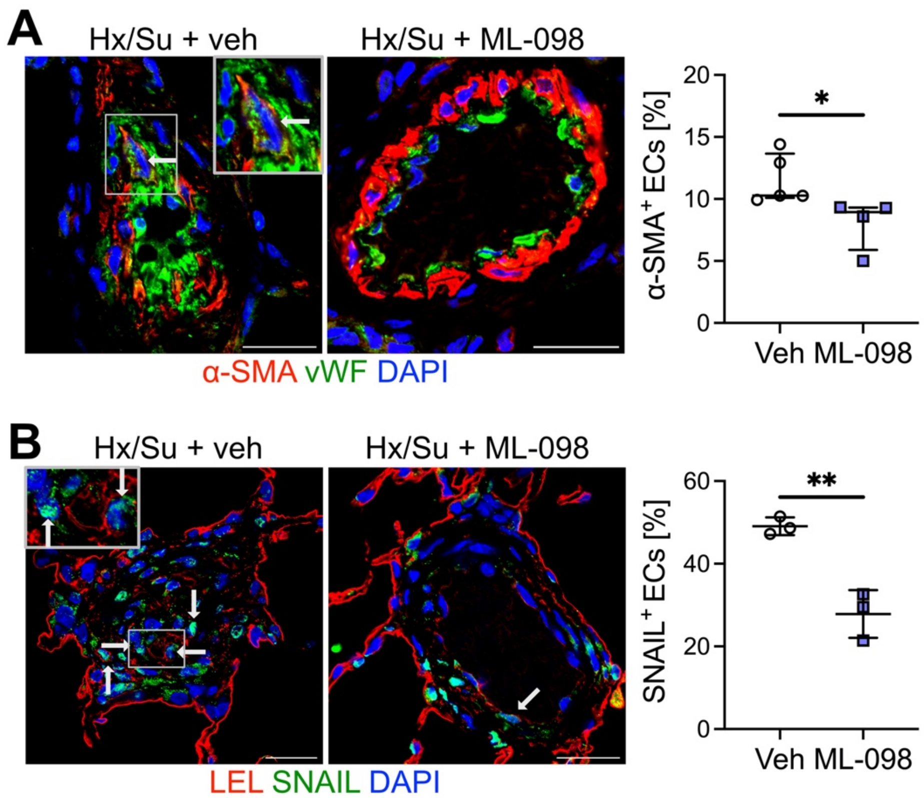

Supplemental Fig. 6. Changes in markers of endothelial-to-mesenchymal transition following interventional treatment with ML-098 (day 22-35) in rats exposed to chronic hypoxia/SU5416. (**A**) Representative optical sections (confocal microscopy) of α-SMA (red pseudocolor) and vWF (green pseudocolor) staining show evidence for endothelial-to-mesenchymal transition (EnMT) by detecting cells expressing endothelial (vWF) and mesenchymal (α-SMA) marker (arrow). The insert shows the cells expressing both markers identified by arrow and dotted rectangle in more detail. The graph shows the quantification of α-SMA$^+$ ECs (α-SMA$^+$ vWF$^+$ cells) in n=4 animals per group. The data are presented as single data points and median±interquartile rank and were compared using the Mann-Whitney rank test. (**B**) Representative optical sections (confocal microscopy) of *Lycopersicon esculentum* lectin-stained cells (LEL, Tomatolectin, red pseudocolor) and SNAIL (green pseudocolor) staining show evidence for the endothelial-to-mesenchymal transition by detecting cells expression endothelial cell marker (LEL) and EnMT transcription factor SNAIL (arrows). The insert shows the cells expressing both markers identified by arrow and dotted rectangle in more detail. The graph shows the quantification of SNAIL$^+$ ECs in n=3 animals per group. For the analysis, ≥10 pulmonary arteries were analyzed for each rat, and the average values for all pulmonary arteries per mouse were used for statistical analysis. The data are presented as single data points and mean ± SD and were compared using a 2-tailed Student's t-test. *P<0.05, **P<0.01. Scale bars: 25 µm.

Supplemental Table 1: Demographics for the patient tissues in Figure 1A.

**Condition**	**Age**	**Gender**
Control 1	54	Female
Control 2	54	Male
Control 3	49	Female
PAH 1	34	Female
PAH 2	7	Male
PAH 3	59	Female

Supplemental Table 2: Demographics for the patient cell lines in Figure 1C.

**Condition**	**Age**	**Gender**
Control 1	49	Female
Control 2	55	Female
Control 3	35	Male
Control 4	61	Female
Control 5	70	Male
Control 6	35	Male
Control 7	34	Female
Control 8	64	Female
Control 9	24	Male
Control 10	47	Male
Control 11	13	Male
Control 12	51	Female
PAH 1	21	Female
PAH 2	31	Male
PAH 3	32	Female
PAH 4	40	Male
PAH 5	50	Female
PAH 6	36	Female
PAH 7	40	Female
PAH 8	55	Male
PAH 9	27	Female
PAH 10	45	Female
PAH 11	32	Male
PAH 12	36	Female
PAH 13	27	Female
PAH 14	16	Male
PAH 15	44	Male

Supplemental Table 3: Demographics for the patient cell lines in Figure 1D.

**Condition**	**Age**	**Gender**
Control 1	32	Female
Control 2	33	Female
Control 3	37	Male
Control 4	40	Male
PAH 1	53	Female
PAH 2	36	Female
PAH 3	31	Male
PAH 4	45	Male

References

1. Kandel J, Chou P, and Eckmann DM. Automated detection of whole-cell mitochondrial motility and its dependence on cytoarchitectural integrity. *Biotechnol Bioeng.* 2015;112(7):1395-405.
2. Jang D, Seeger S, Grady M, Shofer F, and Eckmann D. Mitochondrial dynamics and respiration within cells with increased open pore cytoskeletal meshes. *Biol Open.* 2017;6(12):1831-9.
3. Bhagwani A, Farkas D, Harmon B, Authelet K, Cool C, Kolb M, et al. Clonally selected primitive endothelial cells promote occlusive pulmonary arteriopathy and severe pulmonary hypertension in rats exposed to chronic hypoxia. *Sci Rep.* 2020;10(1):1136.
4. Bhagwani A, Hultman S, Farkas D, Moncayo R, Dandamudi K, Zadu A, et al. Endothelial cells are a source of Nestin expression in Pulmonary Arterial Hypertension. *PLoS One.* 2019;14(3):e0213890.
5. Farkas D, Thompson AAR, Bhagwani AR, Hultman S, Ji H, Kotha N, et al. Toll-like Receptor 3 Is a Therapeutic Target for Pulmonary Hypertension. *Am J Respir Crit Care Med.* 2019;199(2):199-210.
6. Martin M. Cutadapt removes adapter sequences from high-throughput sequencing reads. *2011.* 2011;17(1):3.
7. Andrews S. FastQC: a quality control tool for high throughput sequence data. https://www.bioinformatics.babraham.ac.uk/projects/fastqc/; 2010.
8. Dobin A, Davis CA, Schlesinger F, Drenkow J, Zaleski C, Jha S, et al. STAR: ultrafast universal RNA-seq aligner. *Bioinformatics.* 2013;29(1):15-21.
9. Anders S, Pyl PT, and Huber W. HTSeq—a Python framework to work with high-throughput sequencing data. *Bioinformatics.* 2015;31(2):166-9.
10. Love MI, Huber W, and Anders S. Moderated estimation of fold change and dispersion for RNA-seq data with DESeq2. *Genome Biol.* 2014;15(12):550.
11. Wang L, Wang S, and Li W. RSeQC: quality control of RNA-seq experiments. *Bioinformatics.* 2012;28(16):2184-5.
12. Hennig C. Cran-package fpc. https://cran.r-project.org/web/packages/fpc/index.html.
13. Alexa A, and Rahnenfuhrer J. topGO: Enrichment Analysis for Gene Ontology. R package version 1.38.1; 2019.
14. Mitchell AL, Attwood TK, Babbitt PC, Blum M, Bork P, Bridge A, et al. InterPro in 2019: improving coverage, classification and access to protein sequence annotations. *Nucleic Acids Res.* 2019;47(D1):D351-d60.
15. Geer LY, Marchler-Bauer A, Geer RC, Han L, He J, He S, et al. The NCBI BioSystems database. *Nucleic Acids Res.* 2010;38(Database issue):D492-6.
16. Subramanian A, Tamayo P, Mootha VK, Mukherjee S, Ebert BL, Gillette MA, et al. Gene set enrichment analysis: a knowledge-based approach for interpreting genome-wide expression profiles. *Proc Natl Acad Sci U S A.* 2005;102(43):15545-50.
17. Liberzon A, Subramanian A, Pinchback R, Thorvaldsdóttir H, Tamayo P, and Mesirov JP. Molecular signatures database (MSigDB) 3.0. *Bioinformatics.* 2011;27(12):1739-40.

18. Fabregat A, Jupe S, Matthews L, Sidiropoulos K, Gillespie M, Garapati P, et al. The Reactome Pathway Knowledgebase. *Nucleic Acids Res.* 2018;46(D1):D649-d55.
19. Slenter DN, Kutmon M, Hanspers K, Riutta A, Windsor J, Nunes N, et al. WikiPathways: a multifaceted pathway database bridging metabolomics to other omics research. *Nucleic Acids Res.* 2018;46(D1):D661-d7.
20. Schindelin J, Arganda-Carreras I, Frise E, Kaynig V, Longair M, Pietzsch T, et al. Fiji: an open-source platform for biological-image analysis. *Nat Methods.* 2012;9(7):676-82.
