# Supplementary material for: RAB7 deficiency impairs pulmonary artery endothelial function and promotes pulmonary hypertension
Source: J Clin Invest. 2024 Feb 1;134(3):e169441. doi: 10.1172/JCI169441 (PMC10836802; doi:10.1172/JCI169441)

Full unedited Western blots

RAB7 deficiency impairs pulmonary artery endothelial function and promotes pulmonary hypertension.

Bryce Piper, Srimathi Bogamuwa, Tanvir Hossain, Daniela Farkas, Lorena Rosas, Jose A. Ovando, Adam Green, Geoffrey Newcomb, Nuo Sun, Jeffrey C. Horowitz, Aneel R. Bhagwani, Hu Yang, Tatiana V. Kudryashova, Mauricio Rojas, Ana L. Mora, Pearlly Yan, Rama K. Mallampalli, Elena A. Goncharova, David M. Eckmann, Laszlo Farkas

Full unedited blot for Figure 1B

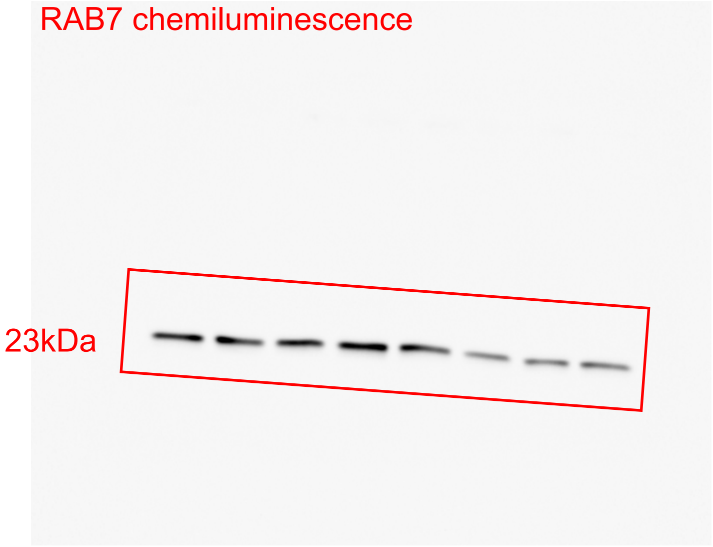

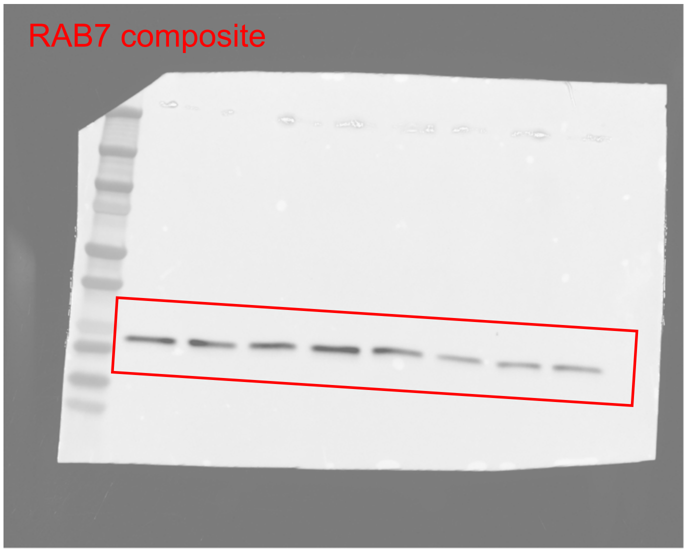

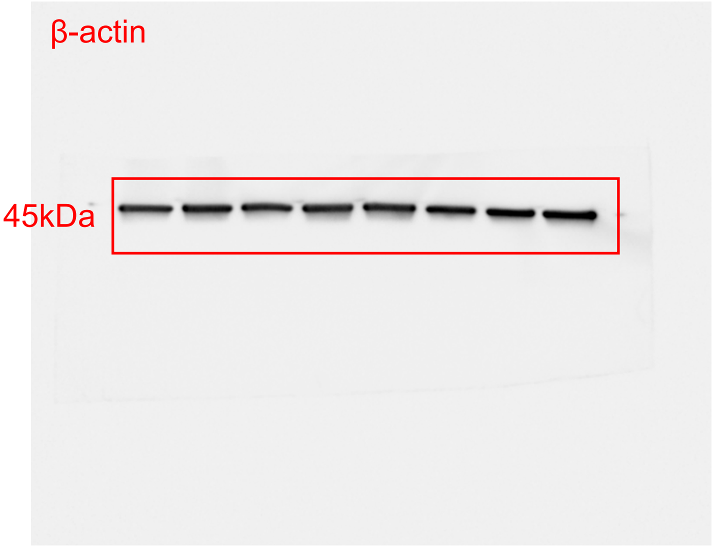

Full unedited blot for Figure 1C

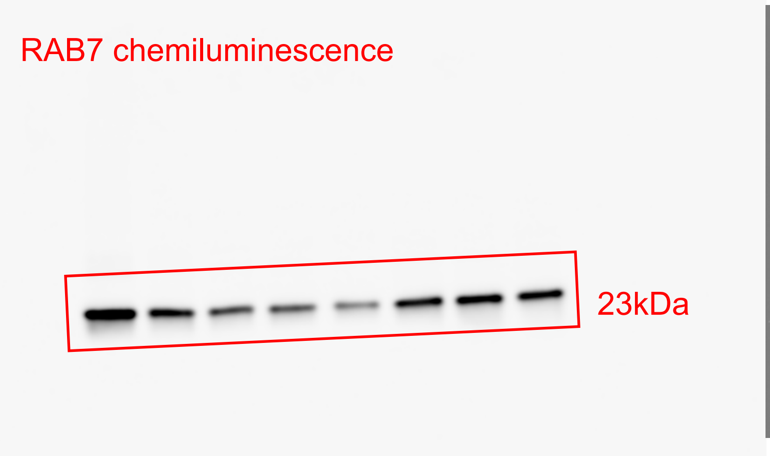

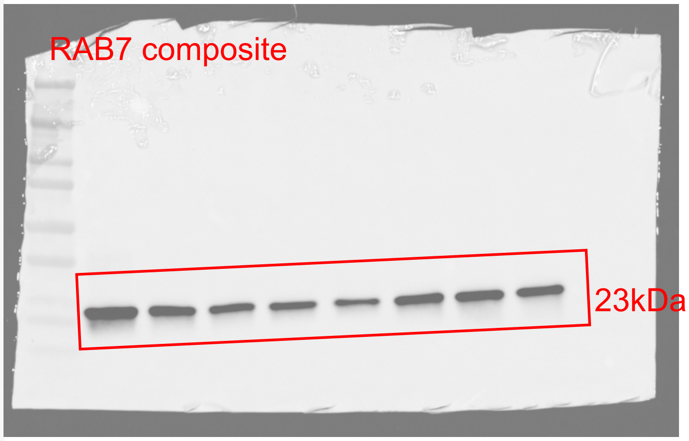

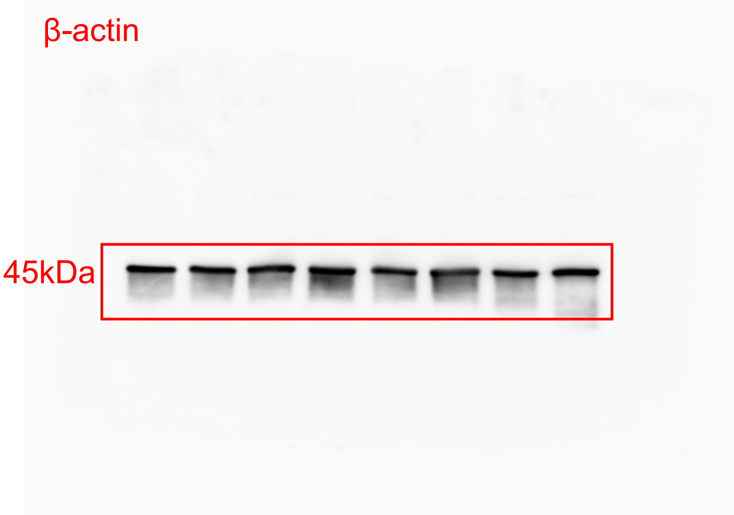

Full unedited blot for Figure 2B

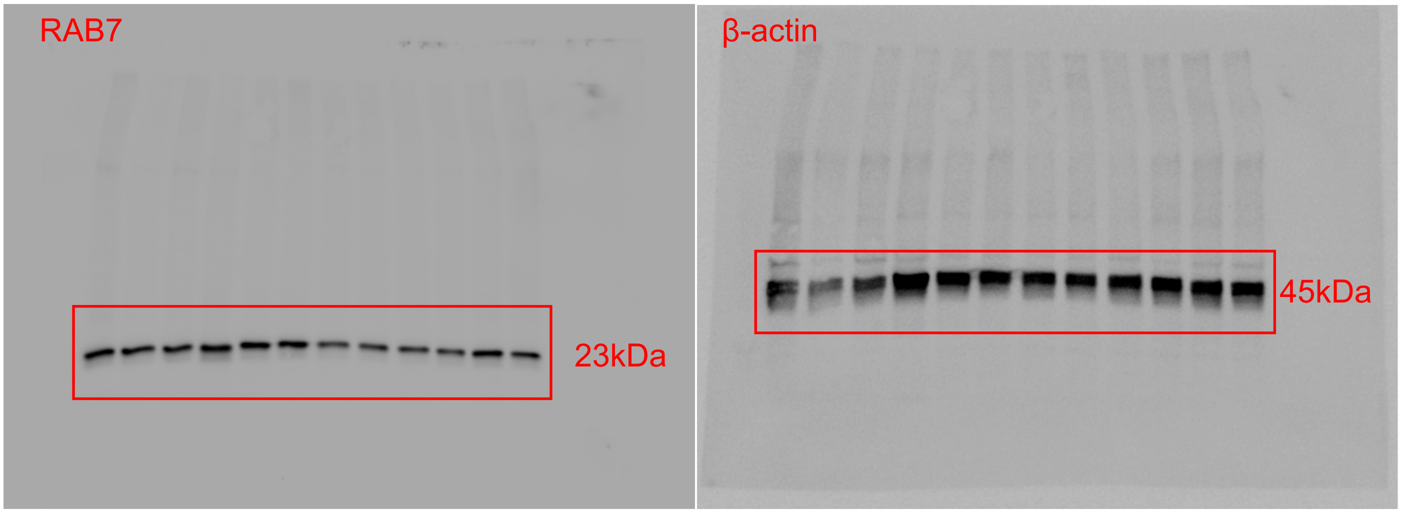

Full unedited blot for Figure 4E

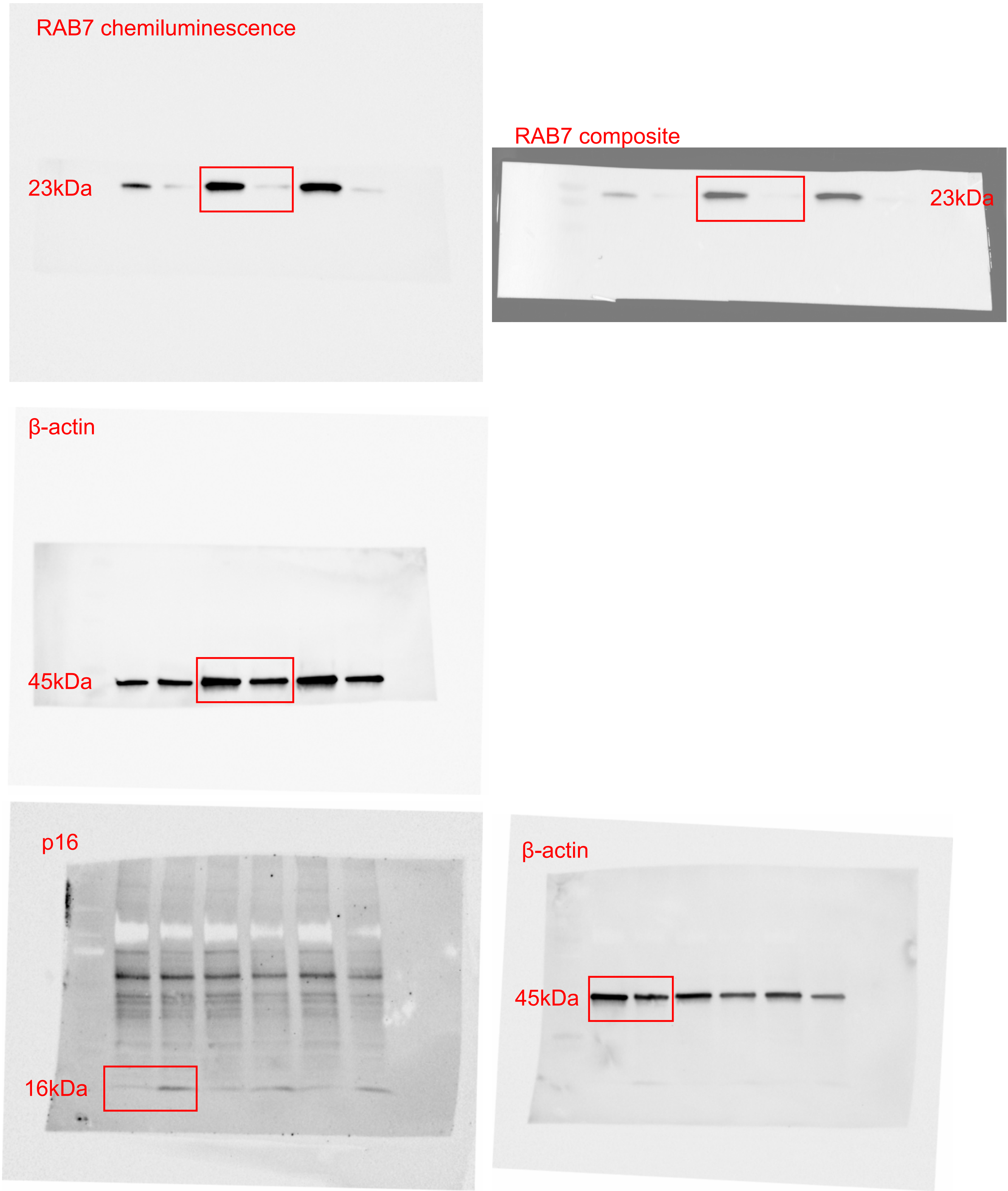

Full unedited blot for Supplemental Figure 4B

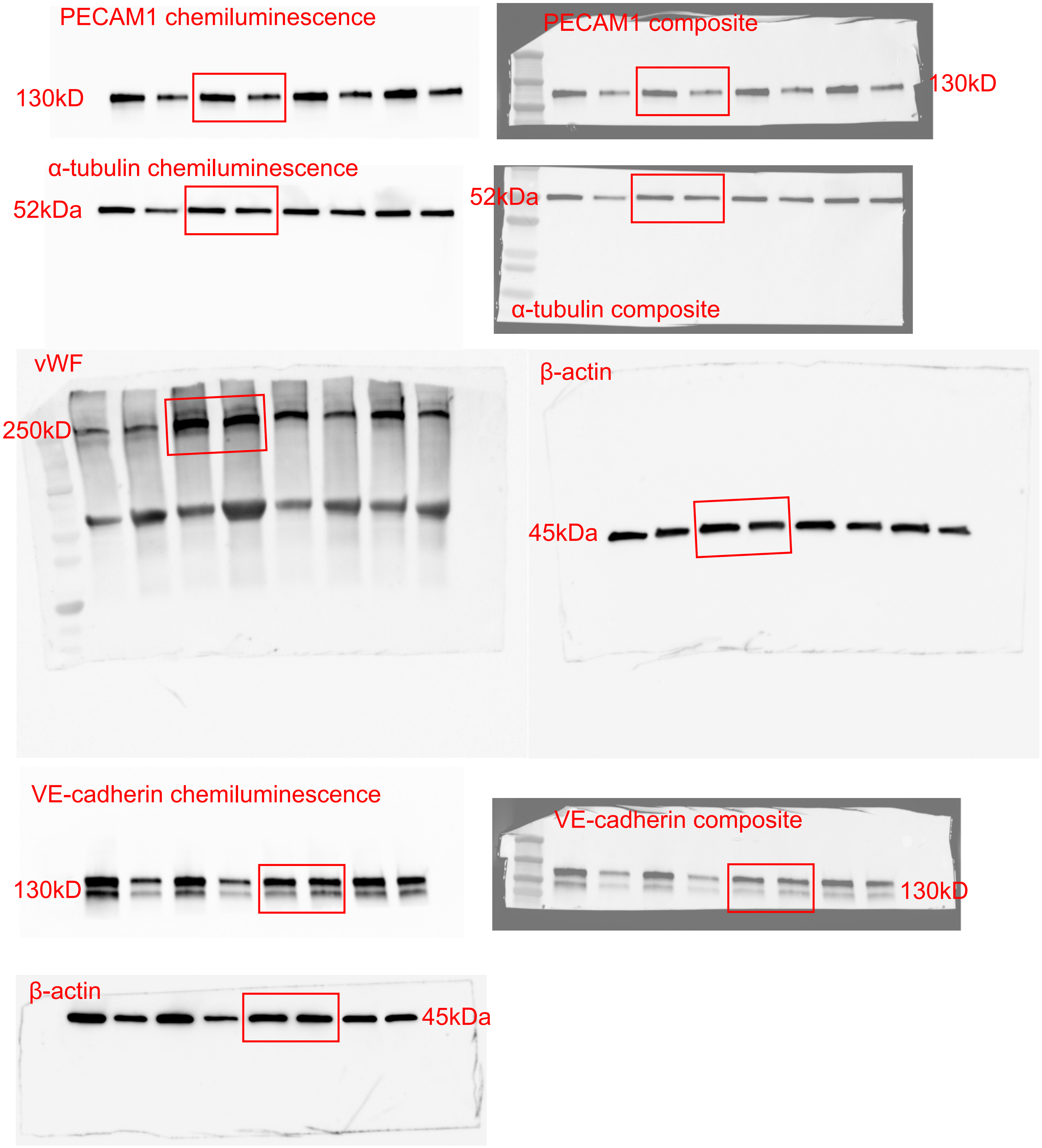

Full unedited blot for Supplemental Figure 4C

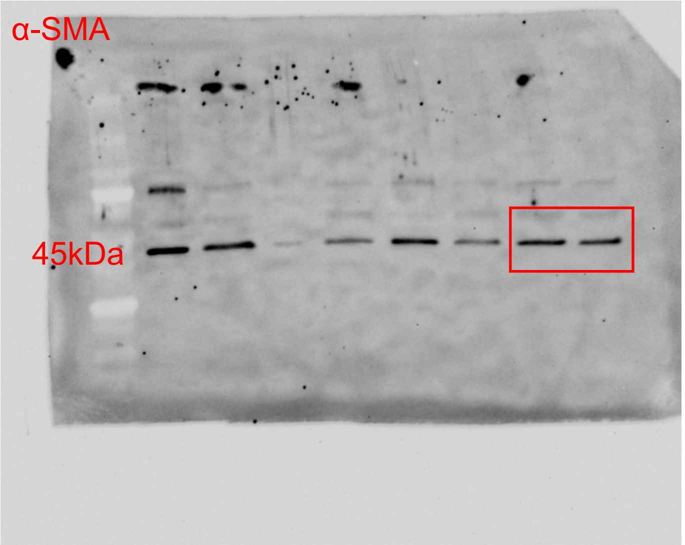

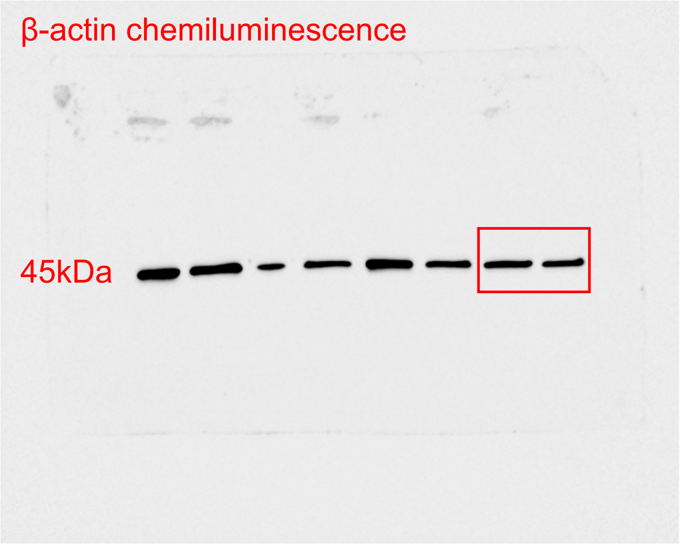

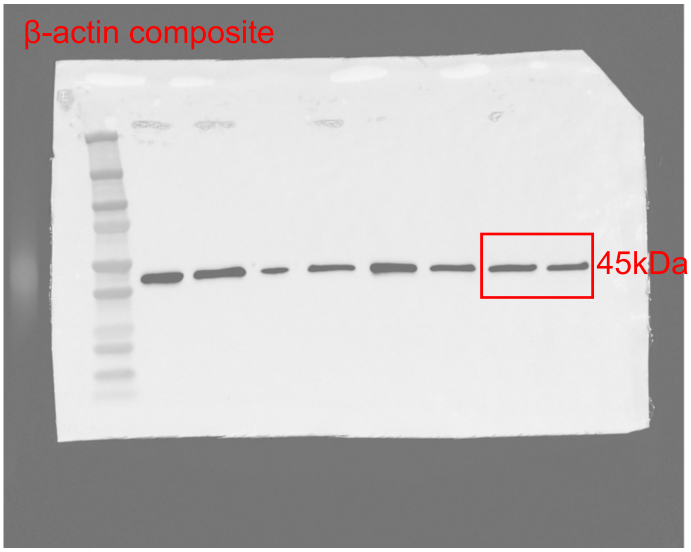

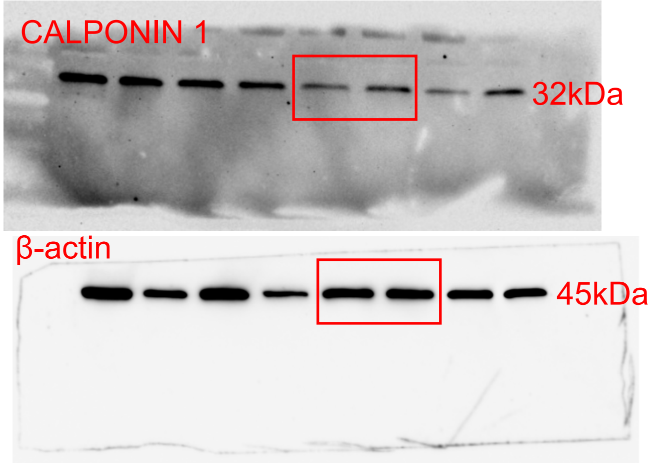

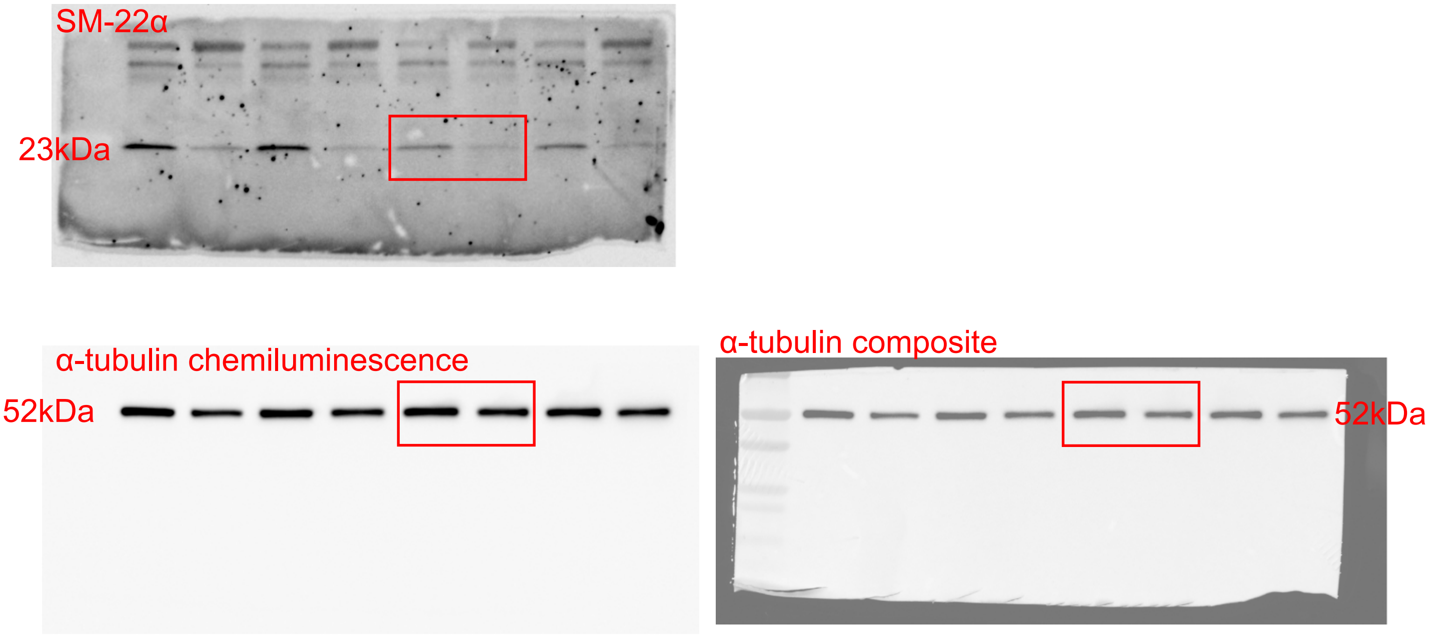
SM-22α
23kDa
α-tubulin chemiluminescence
52kDa
α-tubulin composite
52kDa

Full unedited blot for Supplemental Figure 4D

SNAIL chemiluminescence

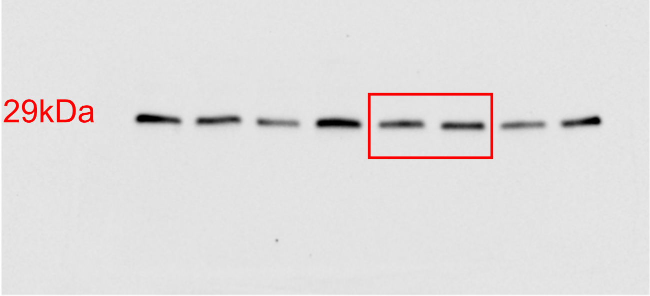

SNAIL composite

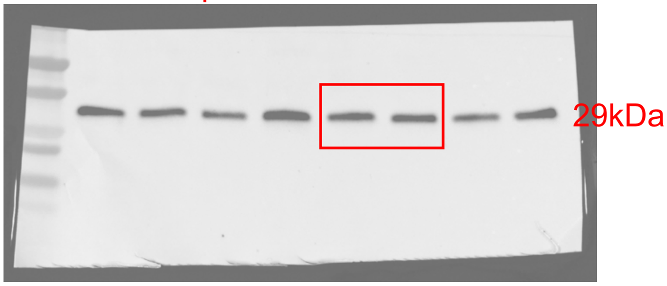

α-tubulin chemiluminescence

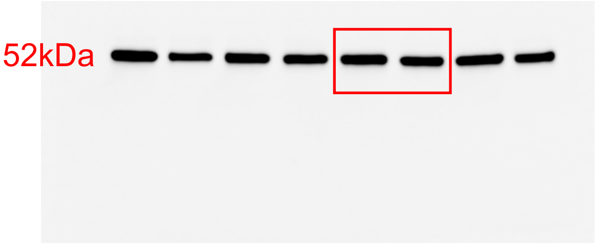

α-tubulin composite

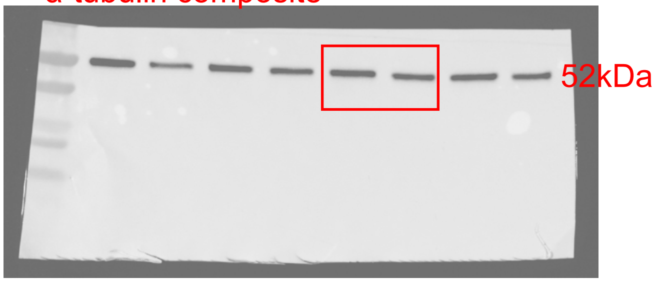

Supplement: Unedited blot and gel images [file jci-134-169441-s103.pdf]
